# Mapping automatic social media information disorder. The role of bots and AI in spreading misleading information in society

**DOI:** 10.1371/journal.pone.0303183

**Published:** 2024-05-31

**Authors:** Andrea Tomassi, Andrea Falegnami, Elpidio Romano

**Affiliations:** Engineering Faculty, Uninettuno International Telematic University, Rome, Italy; Flinders University, AUSTRALIA

## Abstract

This paper presents an analysis on information disorder in social media platforms. The study employed methods such as Natural Language Processing, Topic Modeling, and Knowledge Graph building to gain new insights into the phenomenon of fake news and its impact on critical thinking and knowledge management. The analysis focused on four research questions: 1) the distribution of misinformation, disinformation, and malinformation across different platforms; 2) recurring themes in fake news and their visibility; 3) the role of artificial intelligence as an authoritative and/or spreader agent; and 4) strategies for combating information disorder. The role of AI was highlighted, both as a tool for fact-checking and building truthiness identification bots, and as a potential amplifier of false narratives. Strategies proposed for combating information disorder include improving digital literacy skills and promoting critical thinking among social media users.

## Introduction

**Fig 1:**
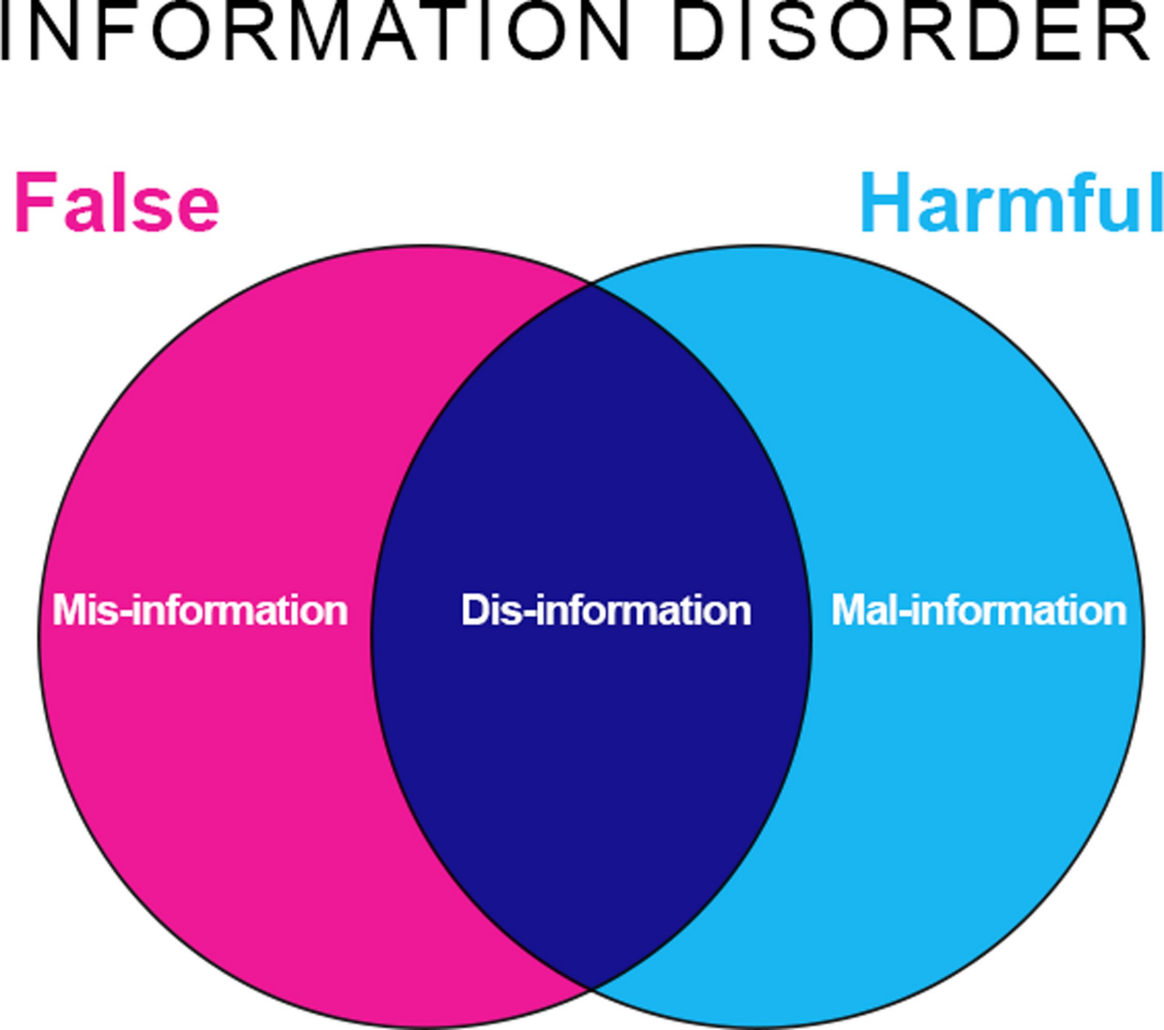
Types of Information Disorder (ID). Adapted from [21].

Social networking platforms such as Facebook, Twitter, Instagram, expose their users to an unprecedented amount of information, where purchase suggestions from recommendation systems, information and opinions from other users, as well as breaking news coexist, which is rather worrying considering the growing importance of social media networks for millions of people worldwide [1–3]. The rise of social media as a source of news and information has been marked by several concurrent phenomena: firstly, the convenience and accessibility of such media facilitate access to news and information from a wide range of sources, generally unverified [4]; the pervasiveness and ubiquity associated with the mode of use (e.g., mobile phone applications) mean that one does not have to wait for the next edition of a newspaper or television program [5]; the underlying social nature of such applications favors the rapid, immediate, and therefore uncontrolled dissemination of content among one’s contacts (both close and acquaintances) and, in a chain, among contacts’ contacts [6]. The well-established phenomenon of homophily (i.e., the tendency to associate among similar individuals) creates online communities that are strengthened by sharing interests, values, and worldviews, amplifying the pervasiveness of ideas that can thus find fertile ground (e.g., viral ideas and memes) [7,8]. While the spread of news and information via social networks has, in some cases, made a significant positive contribution (e.g., Arab Spring, Black Lives Matter, Iranian Women’s Demands for Freedom and similar civil rights uprisings) [6,9–11], many other times there are considerable concerns about the quality and reliability of the information that is shared on these platforms [12,13]. Social media platforms have been widely criticized for their role in spreading misinformation, fake news and disinformation, which can have a significant negative impact on individuals, communities and societies [14,15], as well as for themselves [16]. Although several review works have considered the importance of social media in relation to various phenomena related to the dissemination of untruthful information, to the best of our knowledge it is unclear how these phenomena are distributed over the different existing platforms [17–20]. As social media continue to evolve and play an increasingly central role in the lives of millions of people in an increasingly globalized world, it would be important to create an ideal snapshot of these developments. To avoid confusion, we need to clarify the differences between the various Information Disorders (ID) that may appear very similar at first glance (Fig 1) [21].

“Misinformation”: incorrect information disseminated without intent to deceive or harm a third party; “Disinformation”: intentional dissemination of manipulated and/or false information with the specific intent to harm and manipulate someone; “Malinformation”: real information ‐ but presented in a distorted manner ‐ used for the purpose of harming or manipulating the judgement of others [22,23].

It is essential to identify the different actors behind the dissemination of false or harmful information, their motivations, and the methods they use [24,25]. The various social media platforms have unique characteristics that make them more susceptible to misinformation, disinformation and malinformation and this should be taken into account when designing interventions to mitigate their spread.

Moreover, the significant advancement of Artificial Intelligence has multiplied the complexity and multifaceted nature of the problem of source verifiability by several orders of magnitude [26–28]. From an ontological perspective, deception is a fundamental characteristic associated with human intelligence. For this reason, in the inability to define exactly what intelligence is, the Turing test was created to evaluate whether a machine can be considered intelligent and is based on the verisimilar interaction between humans and computers [29,30]. The test only verifies whether the machine is able to dissimulate itself in a credible and convincing manner, as a human would [31]. In this sense, deception can be considered the "original sin" of A.I. It is humans who project humanity and intelligence onto machines that appear to possess similar abilities to ours, stimulating authentic empathy and, sometimes, authority. For example, it is important to carefully consider the ease and speed with which a cyber-sociotechnical agent, like a conversational Bot, can generate seemingly valid content [32]. A.I. is generating new opportunities to create or manipulate texts and images, audio or video content [33]. Moreover, A.I. systems developed and deployed by online platforms to enhance their users’ engagement significantly contribute to the effective and rapid dissemination of disinformation online [34]. Finally, specific bots connected to social network platforms might be designed with the aim of acting as fake-news super-spreaders [35].

In such a world where information can be easily accessed, evaluated, and disseminated on an unprecedented scale, individuals must therefore possess the necessary skills to assess the credibility of sources and the content they encounter [36]. Critical thinking plays a crucial role in the fight against disinformation, malinformation, and misinformation on social media platforms [37]. Critical thinking enables the identification of logical fallacies, the evaluation of evidence and the validity and reliability of claims [38]. By cultivating critical thinking skills, individuals can more effectively identify and avoid false, misleading, or manipulative information on social media platforms, reducing the risk of falling prey to disinformation, malinformation, and misinformation [39–42].

Considering the above, this review aims to identify new insights into the phenomenon of fake news on social networking platforms. Specifically, addressing:This raises ethical and cultural questions about the need for interdisciplinary reflection to address these dynamics.

1. How does misinformation, disinformation, and malinformation distribute across different social media platforms?
2. What are the recurring themes in fake news? On which platforms do they find greater visibility?
3. How does artificial intelligence relate to the issue of fake news? As an authoritative agent or as a spreader agent on social networks?
4. What is the role of Critical Thinking as identified in the scientific literature related to the investigated problem?

The remainder of the article is developed as follows: the next section outlines the methodologies used; section 3 sets out the findings discussed in section 4, of which part 4.4 draws conclusions, limitations and future developments of this work.

## Methods

The research team (consisting of: 2 psychologists experienced in Critical Thinking Assessment; 2 psychologists experienced in the construction of cognitive-behavioural models; 2 engineers versed in computational document management in complex socio-technical systems; 2 engineers experienced in network analysis; 1 engineer experienced in social network platforms) sought to take an agnostic approach to the distribution of ID and related topics on social media, as will be detailed in the following paragraphs. More in detail, after a preliminary focus-group, the team developed the general idea that relationships between ID themes and social network platforms could be identified as emerging categories from relevant documents drawn from existing literature [43]. Team’s multidisciplinary resulted to be essential in the retrieval and screening stages as well as during the validation one. The data analysis was performed by the engineers while the entire team worked on interpretation of results.

**Fig 2:**
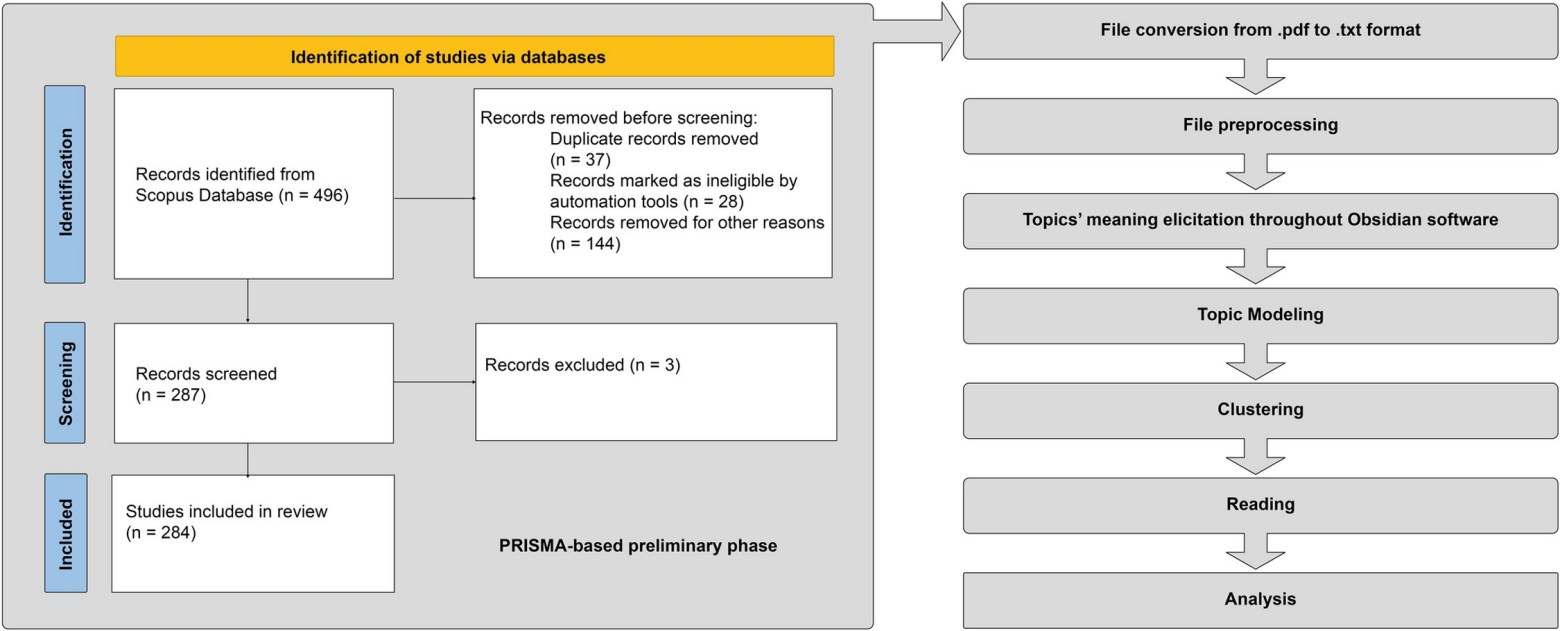
The PRISMA workflow. Left panel shows the Prisma part of the literature review. Right panel reports the processing part of the workflow.

The core concept behind this proposition is that scholarly articles pertaining to specific platforms ought to encompass comprehensive discussions on relevant ID subjects as well. The less stringent the search query, the larger and more statistically valid the documentary sample that will form the basis for concept extraction. Alongside the latter consideration, the team also attempted to design a practicable methodology workflow (Fig 2).

### Primary sources selection and extraction of articles

The research team identified the Scopus scientific database as a trustworthy and adequate source of articles to answer the research questions in this enquiry. Indeed, Scopus covers over 76 million records of scientific articles published by over 40,000 publishers worldwide, although it is important to note that Scopus does not cover all existing scientific journals, but only a selection of those considered to be of high quality and scholarly relevance. Some estimates suggest that Scopus coverage is over 86%. While aware that Scopus does not encompass the entirety of existing scientific journals, the decision to rely exclusively on this database was driven by a thorough evaluation of its coverage and representativeness in our specific research field. Most seminal works and leading studies within our area of interest are included in Scopus, which indicates that the percentage of potentially omitted research is significantly low [44,45]. Consequently, we maintain that despite this limitation, the robustness and validity of our findings remain intact, accurately reflecting current trends and significant discoveries in the field of study.

In our literature review methodology, we prioritized both the relevance and reliability of the sources through meticulous adherence to the PRISMA protocol and the exclusive use of the Scopus database. By employing the PRISMA protocol, we ensured a systematic, transparent, and rigorous approach to selecting studies directly related to our research objectives, thereby upholding the relevance criterion. Simultaneously, the reliance on Scopus guaranteed the inclusion of only peer-reviewed publications, affirming the reliability and scholarly merit of our sources. This dual emphasis on the PRISMA protocol and Scopus’s peer review process underscored our commitment to basing our review on literature that is both directly pertinent to our study and of verified quality, thereby reinforcing the credibility and robustness of our findings.

The search query submitted to the Scopus engine on 10 May 2023 included original articles and journal reviews in English, with no date restrictions (i.e., from the very first publication about the topic queried to 10 May 2023). In more detail, the query sought to identify in texts all possible declinations of the terms ‘misinformation’, ‘disinformation’, ‘malinformation’, and ‘fake news’ no more than five words away from the name of one of today’s most relevant social networks. The query is reported below:

TITLE-ABS-KEY((disinformation OR misinformation OR "fakenews" OR malinformation) AND (("socialnetwork" OR media OR platform) W/5 (facebook OR twitter OR instagram OR whatsapp OR youtube OR "TikTok" OR linkedin OR telegram OR wechat OR douyin OR snapchat OR kuaishou OR vkontakte OR "Sina Weibo" OR odnoklassniki OR livejournal OR "Moi Mir"))AND NOT(tv OR newspaper OR radio)) AND (LIMIT-TO(DOCTYPE,"ar") OR LIMIT-TO(DOCTYPE,"re")) AND (LIMIT-TO(LANGUAGE,"English"))

**Fig 3:**
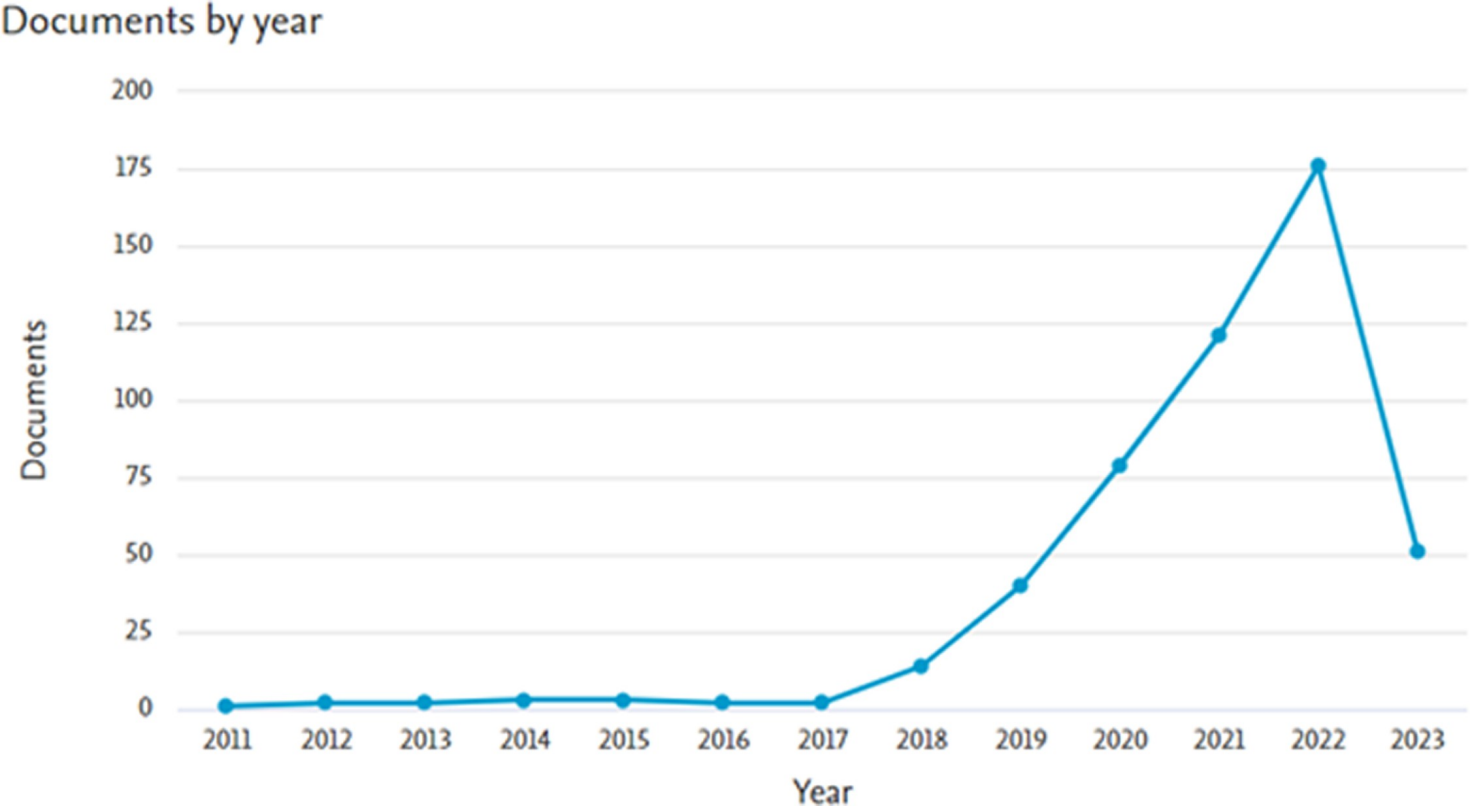
Evolution over time of academic production. Misinformation, disinformation, and fake-news are investigated collectively.

The query returned 496 documents in total. Fig 3 depicts the evolution over time of scholarly publications attempting to establish the phenomenon of ID through the different social networking platforms.

It is worth noting how scholarly production greatly accelerates after 2017, despite the major social networking platforms were born more than 10 years earlier. The steep increase in production, consequence of an increased interest in the subject, might be related to a combination of both cultural and social modification (e.g., a change in consumption by news users which abandoned traditional media as newspapers, magazines, radio and television broadcasts) and epochal historical events (e.g., Brexit major vote outcome in 2016, Trump’s election in 2017, pandemic outbreak in 2019, as well as 35 terrorist attacks).

**Fig 4:**
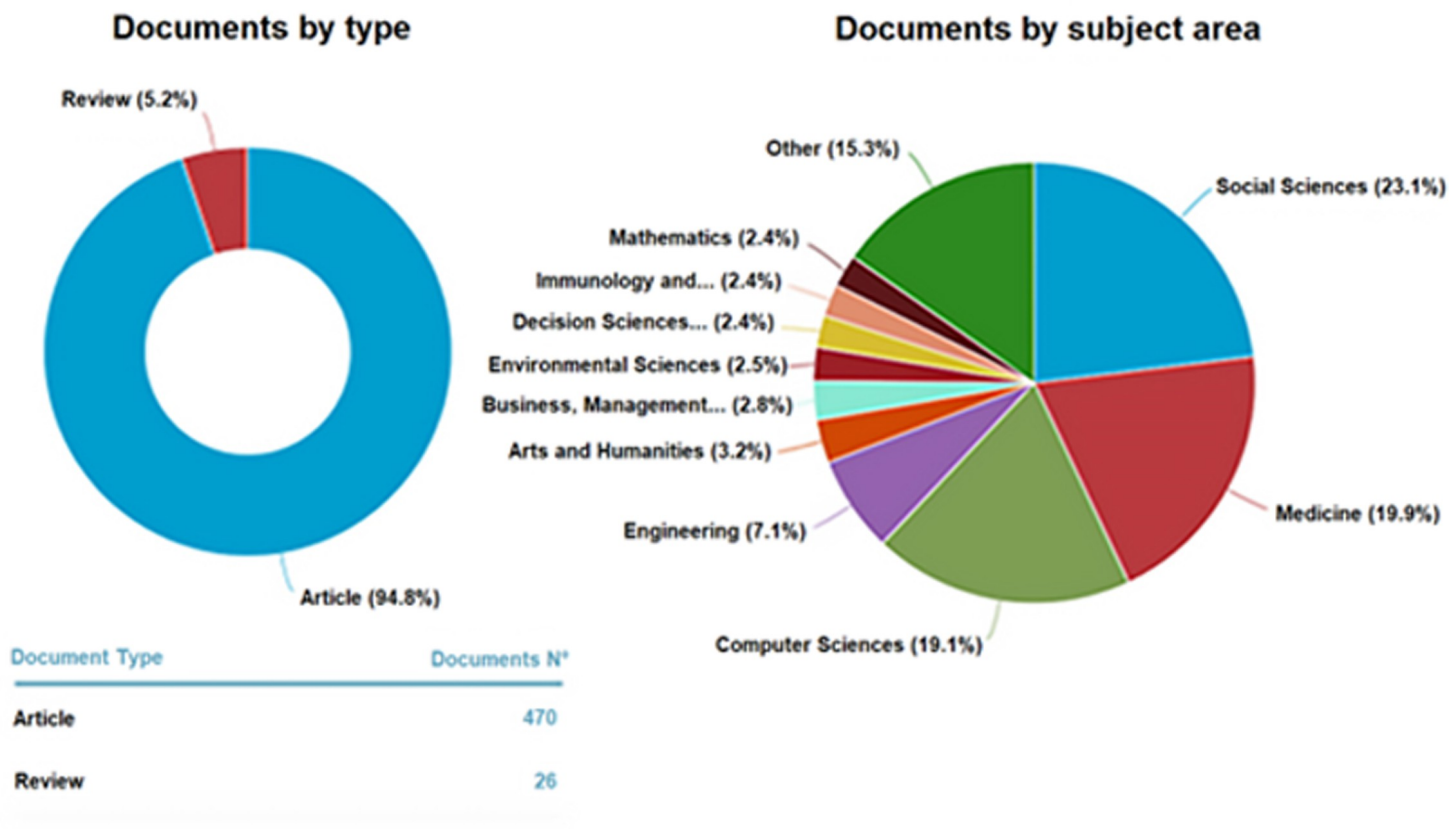
Stratification of the documents collected. Amount of documents analyzed per type (left panel). 94.8% Original Articles vs. 5.2% Reviews; Percentage of subject area to which the articles pertain (right panel).

Drawing general interpretations at this stage of the analysis is not meaningful, given the potentially biased sample. In this regard, Fig 4 points out that articles and journal reviews were the primary sources chosen, excluding conference papers and reviews ‐ notoriously shorter-lived but more capable of capturing the immediacy of events ‐ and books ‐ generally texts of deeper and more thoughtful reflection.

**Fig 5:**
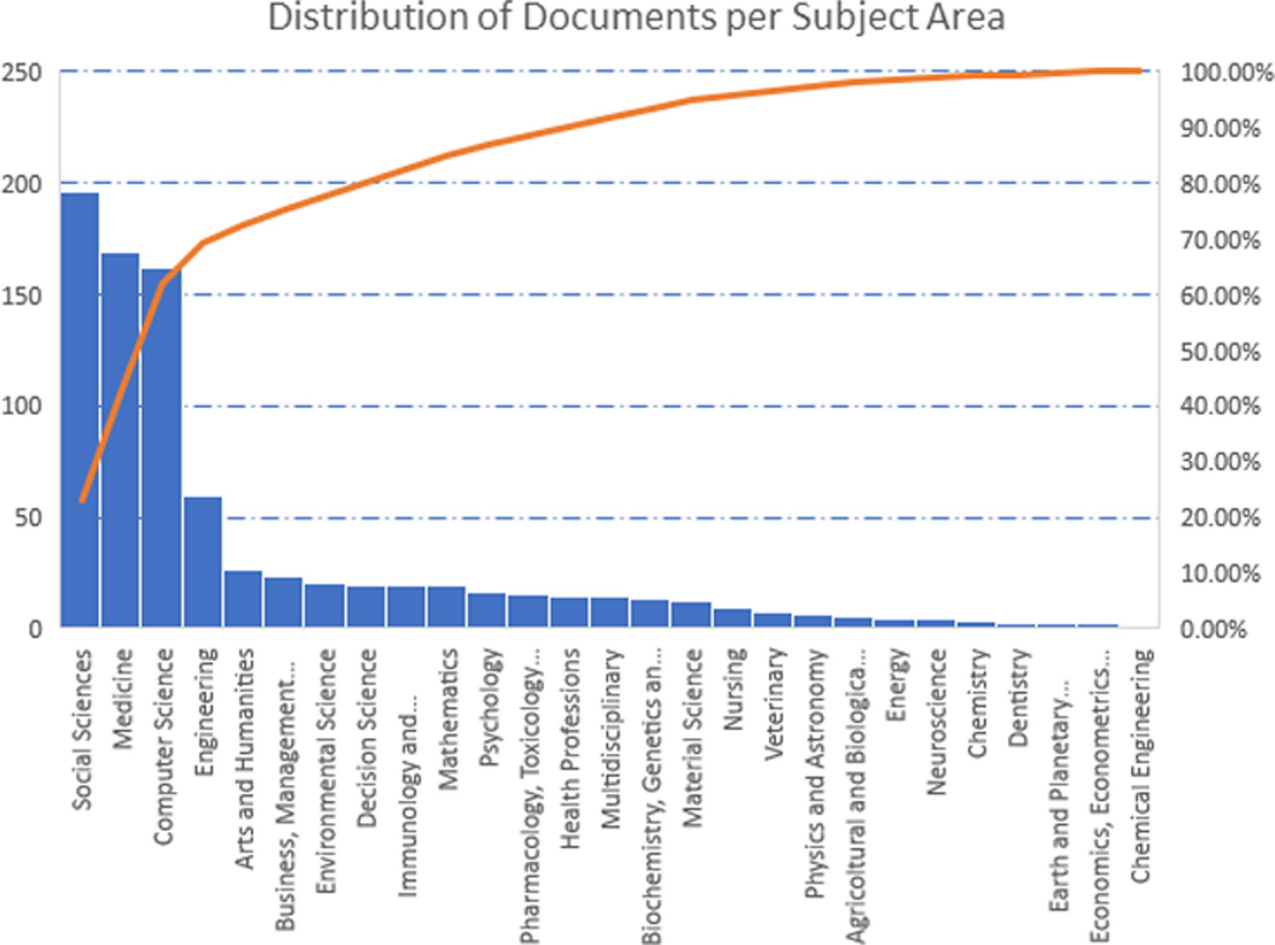
Documents distribution per subject area. How highlighted by Pareto diagram, ID topics are addressed for the most (∼ 70%) by four areas: Social Science (196 papers), Medicine (169), Computer Science (162), and Engineering (60).

While caution should be necessary, the distribution of interest in each of the subject areas is shown (Fig 4, right panel). Analogously distribution of documents per subject area (Fig 5) clearly captures the importance of political and health issues that have been pressing in recent years for the global landscape, which is confirmed by the results of the analysis (cfr. §3.2).

### Retrieval of actually available documents

The identified potential pool of documents from the previous stage has been downloaded, then 352 articles out of the 496 have been retrieved. Such a loss is due to the fact that only open access or subscripted documents can be accessed. After removing duplicates, the actual number dropped further to 287, accounting for the 58% of the original corpus, in other word the final loss stands at 42%.

### File conversion from pdf to txt format

The final graphic format adopted by individual journals in PDF format is ideal for human reading, but completely unsuitable for computational linguists or automatic text interpretation software. A library for the R language was used for this purpose, which is one of the most efficient ways for converting PDF files to txt files [46]. After this stage, a document matrix has been created, where each row represents a document within the corpus and columns bring important information, e.g., document identifier code, name of the corresponding txt file, authors, title, and most important, a column containing the whole paper text, allowing for subsequent natural language processing (NLP) tasks.

### File preprocessing

The parsing of text from pdfs downstream of the previously described step must be considered partial, as various residual pdf structures such as headers, page numbers, XML definitions, figure references, notes, cross-references and graphic frames remain. These translation ‘artefacts’ are not the only textual elements to be removed to enable the subsequent natural language analysis steps. Rather standardized text processing is indeed required.

#### Lower-casing

The text is changed to lower-case for purposes of uniformity promoting faster comparability and consistency across the papers being analyzed. When the text is presented in a consistent lower-case format, the algorithm can focus solely on analyzing the content and semantic patterns without being influenced or misled by variations in capitalization. This standardized approach simplifies the computational and linguistic processing involved in a more efficient tokenization (cfr. § 2.4.4), word normalization, and language modeling techniques reducing the complexity of these tasks and enhancing performance and accuracy.

#### Stop words removal

As is well known, a text is not a random sequence of words, which is why there are words in every language that are much more frequent than others [47,48]. Such words are mostly connectors and articles, terms that serve the correct syntactic and morphological construction of the sentence but do not contribute to the semantic content. Other words that also do not bring semantics to the text are all those that are part of the jargon of scientific journals such as “authors”, “methods”, “lsevier”, “springer”, “results”, “figure”, “table”, etc. All these combined constitute the set of *stop words*, that is words to be ignored when processing text.

#### Removal of numeric and punctuation

Likewise stop words, punctuation marks and numbers are also uninformative when it comes to discerning content themes in texts.

#### Tokenization

Once irrelevant information has been eliminated, tokenization can begin. It refers to the process of breaking down a text into individual tokens or words, which can then be analyzed and processed by a computer program. Tokenization involves identifying the boundaries between words in a text and assigning each word its own unique identifier, known as a token [49].

#### Lemmatization

Lemmatization is the process of reducing a word to its base form (i.e., root). It involves taking away any inflectional suffix or prefix from a word to obtain its simplest and most basic form, making it easier to compare and analyze them across different documents [49]. At this point, the document matrix becomes a document × term matrix, where each row represents a document, and each column a unique word in the entire corpus. The cells of the matrix contain the frequency or presence/absence information of each word in each document. That is the starting point for representing the corpus and its documents as numeric vectors and, therefore, allowing several machine learning techniques, e.g., clustering.

### Clustering

**Fig 6:**
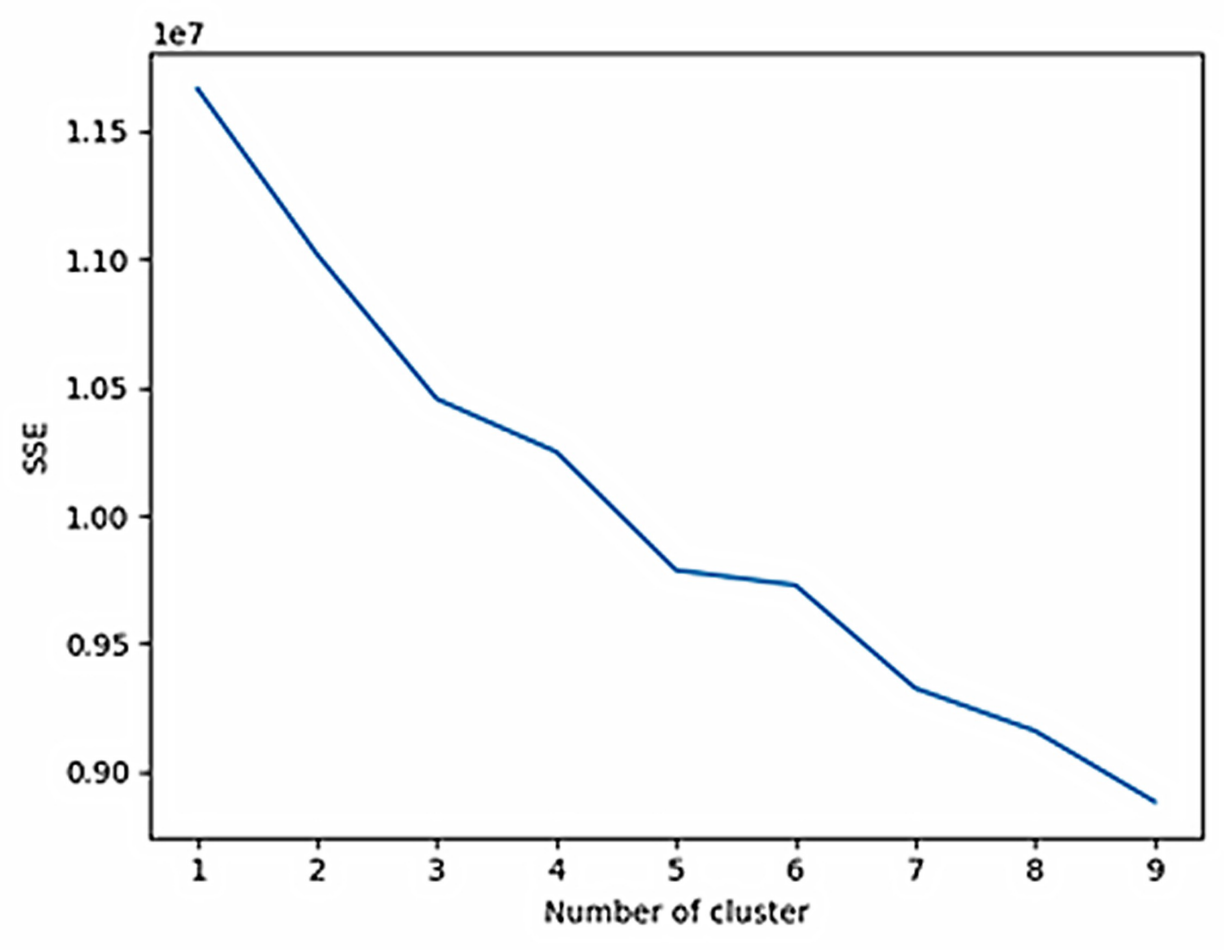
Line plot implementing the elbow method for our corpus. Sum of Squared Errors (SSE) vs Number of clusters. The absence of any clear elbow means that further explorative evaluations are needed.

*Document clustering* focuses on identifying groups of related papers with similar content. This process relies on feature extraction techniques to represent each document’s content in a numerical vector space model. The resulting vectors capture the semantic meaning of each document and allow algorithms to calculate similarities between them. The idea is that, once grouped together, these documents can reveal important information about the overall structure and organization of the documental corpus obtained during the retrieval stage (§ 2.2). The clustering stage helped to focus on key areas of research more efficiently than sifting through every single paper individually. Fig 6 shows the implementation of the *elbow method* for the corpus retrieved [50,51]. The line plots the Sum of Squared Errors (SSE) versus the number of clusters.

SSE calculates the sum of the squared Euclidean distances between each data point and its centroid within a cluster, quantifying the compactness of the clusters, with lower values indicating tighter and more well-defined clusters. In the elbow method, the SSE is often plotted against the number of clusters *k*, and the “elbow” point represents the optimal *k* value (adding more clusters does not significantly decrease the SSE), but the line does not show any clear elbow. Therefore, while the elbow method provides a useful heuristic for determining the number of clusters, it should not be solely relied upon: other factors such as domain knowledge, interpretability, and practical considerations should be considered when deciding on the final number of clusters. In the present case, through repeated analyses of the significance of the emerged topics, using a trial-and-error approach, we managed to identify 6 clusters/themes– 5 meaningful and the remaining one accounting for the “others” category. The identified 6 clusters will represent the potential topics to be identified in the following topic modeling stage.

### Topic modeling

**Fig 7:**
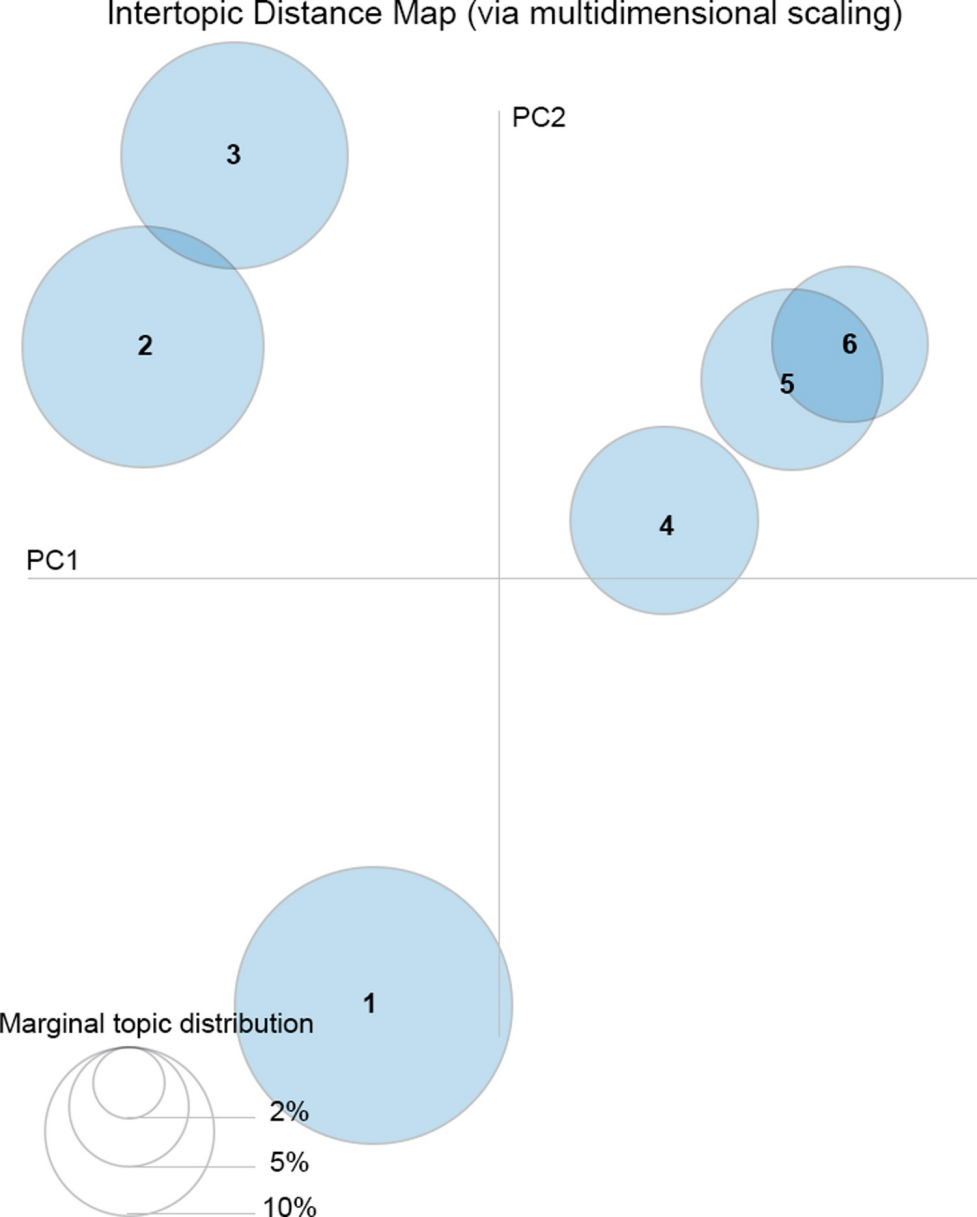
LDA algorithm results. The topics projection on the most informative subspace (ℝ^2^) of the space derived from the Principal Components; the size of the circles is proportional to the marginal topic distribution (i.e., the number of words/terms covered by the topic).

In this stage the research team implemented Latent Dirichlet Allocation (LDA), a generative probabilistic model assuming each document in the corpus as a mixture of a few latent topics, and each topic is characterized by a distribution of words. The number of the latent topics must be known in advance to apply LDA, and then the clustering phase. Then, an algorithm iteratively assigns words to topics and topics to documents based on statistical distributions, aiming at finding the optimal topic-word assignments that best explain the observed numeric data. The latter stage is the so-called “model training”, after which the results can be analyzed. This includes examining the topic-word distributions, which show the probability of each word belonging to a particular topic (i.e., cluster). Fig 7 gives an informative insight into topics distribution.

The corpus whole information has been translated into a numeric format, and the entire document-word matrix define a vector space of cardinality as large as the rank of the matrix. The eigenvectors of such a matrix can be thought as components in which information principally distributes. By choosing a subspace as much informative as possible, the in-between conceptual distance among the topics can be visually represented.

**Fig 8:**
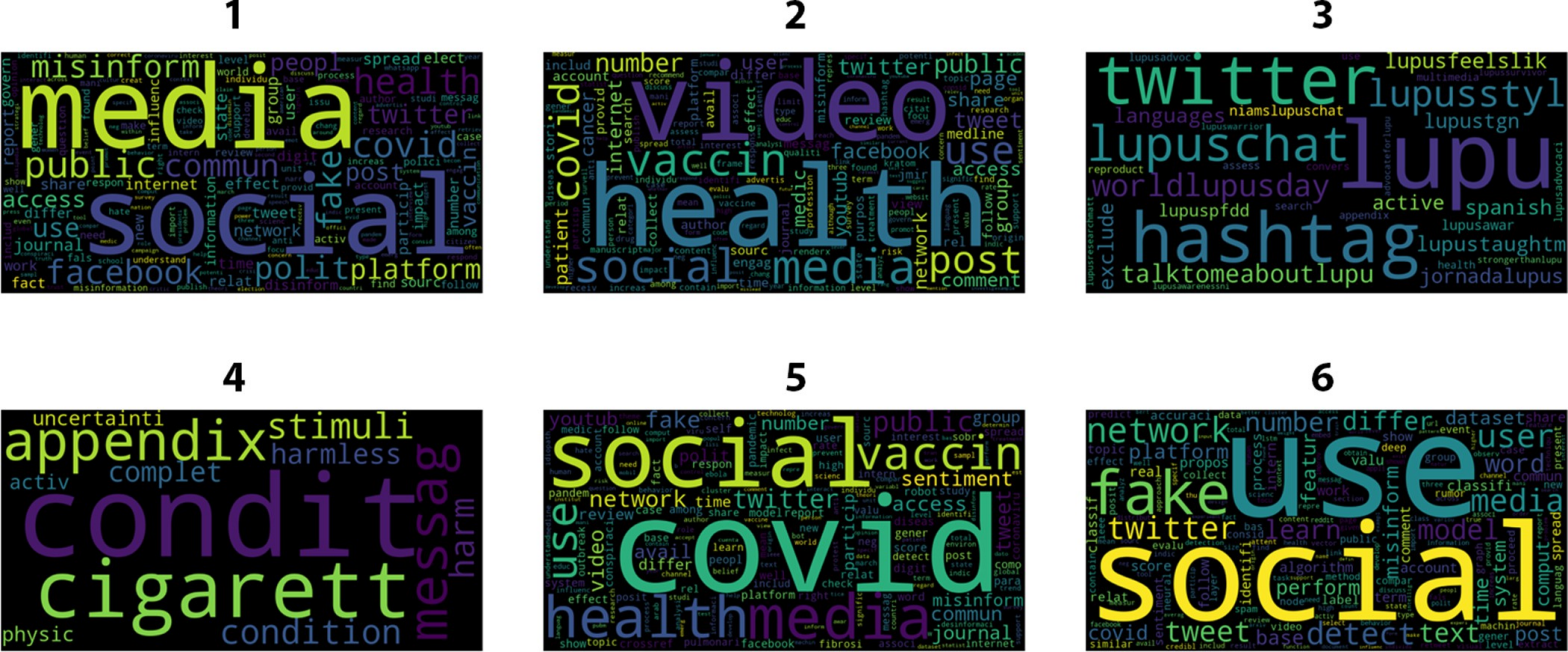
Bag-of-words relative to the six clusters identified. Note a certain degree of terms and topics overlapping, reflecting the unclear behavior of the line plotted for the elbow method.

LDA assigns a probability distribution of topics to each document in the corpus. This allocation allows researchers to understand the primary topics present in individual documents and analyze the document-topic relationships. To interpret the topics, we have analyzed the most probable words associated with each topic and then we were able to infer the underlying theme or meaning of each topic. The final identification of these meanings took place during a face-to-face discussion in a focus group of all the researchers, which ended when consensus was reached (Fig 8).

In this way it becomes possible to uncover hidden thematic structures in the next steps, and in the end understand the content of the retrieved corpus of local text files in a more systematic and objective manner.

### Topics’ meaning elicitation throughout Obsidian software

**Fig 9:**
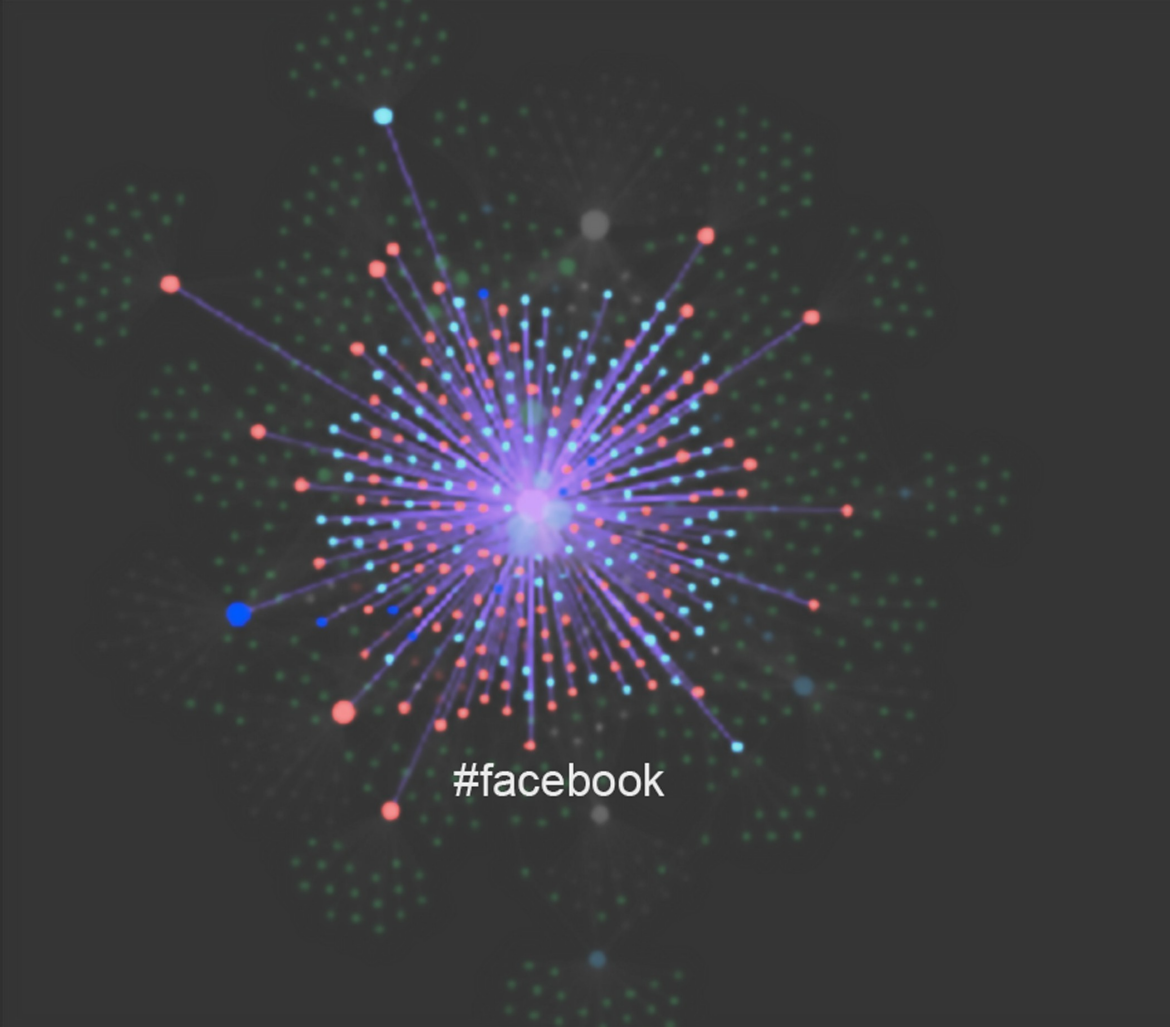
The documental corpus in an initial stage of the topic elicitation through the Obsidian software. Note the cluster around tag #facebook formed by red nodes (papers containing the word “facebook”) and light-blue nodes (papers containing the word "twitter”).

To structure the process of topic identification and unlock other potential information investigation techniques, the corpus document files have been imported into Obsidian software, a powerful markdown interpreter. The documents, now in .md format, are treated within the software as "notes," which in the ordinary intent of the software constitute the atomic elements of a knowledge management system. In Obsidian, each node is identified by a name (in the use case derived from the filename) and relationships between notes are set by using tags or direct links. A word preceded by the hash symbol # turns it into a tag, while the name of a destination note within double square brackets in the body of the originating note defines a direct link. Once these links are created, it is possible to represent the collection of notes as a network or, more appropriately, as a *knowledge graph* (Fig 9).

**Fig 10:**
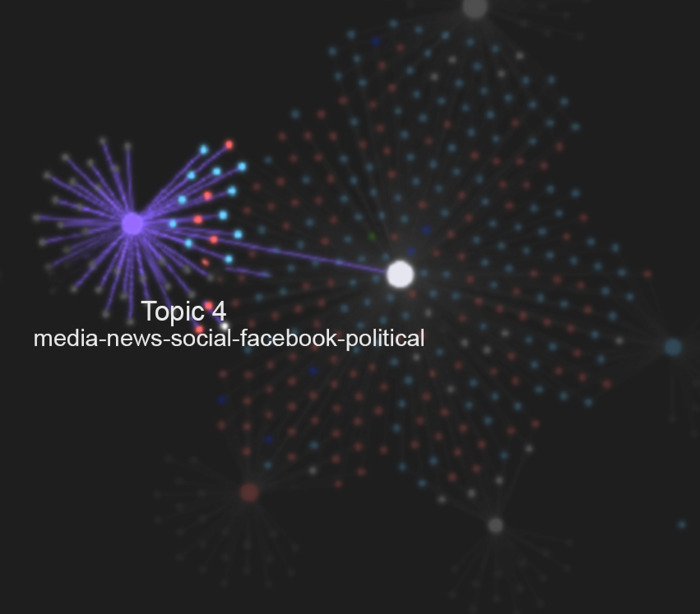
The birth of topic 4. The newly born Topic 4 emerges during the initial phase of topic elicitation in the knowledge graph.

This feature, along with the numerous free plugins developed by the thriving and active user community, makes this software tool versatile and particularly powerful for structuring and retrieving information, as well as eliciting knowledge. This tool has already been tested in several academic research projects, but it is the first time it is being used for the elicitation of predefined topics in a corpus of documents. In this case, indeed, the topics are defined by the research questions presented in the introductory section. Therefore, we are interested in knowing:

- Which documents cite different social networking platforms or, from a specular perspective, given a specific social network (e.g., Twitter), how it gathers certain documents rather than others.
- Given a *bag-of-words* (the words that cluster around a topic), which documents contribute to its saturation. This reasoning translates the fact that the topic emerges from the recurrence of semantically related themes as they are distributed within the corpus of documents. In this context, it is possible to define the "topic" note as the node in the graph that acts as a broker between the words in the bag-of-words and the documents reflecting it. Essentially, the topic note is the note that points to all those documents containing the terms of the corresponding bag-of-words (Fig 10).
- The previous point makes it possible to directly define the literature matrix (the document-feature matrix of the literature analysis) based on the graph analysis. In fact, the adjacency matrix of this new graph, in which both documents and "topic" nodes appear, corresponds precisely to the literature matrix of the investigated corpus.

### Reading

Finally, the documents are read and analyzed, and the literature matrix is validated.

## Findings

After the screening stage, the corpus includes 283 documents (listed in Table 1) that have been converted into Obsidian notes. That allowed for both knowledge graph construction and the implementation of advanced text search tools (e.g., regular expressions pattern matching is natively implemented in Obsidian) over the whole corpus.

**Table 1:** Complete list of the screened documents.

ID	Document Reference	Title
**1**	[52]	Awareness toward COVID-19 precautions among different levels of dental students in King Saud university, Riyadh, Saudi Arabia
**2**	[53]	Examining algorithmic biases in YouTube’s recommendations of vaccine videos
**3**	[54]	Impact of public sentiments on the transmission of COVID-19 across a geographical gradient
**4**	[55]	Arabic rumor detection: A comparative study
**5**	[56]	Are people incidentally exposed to news on social media? A comparative analysis
**6**	[57]	Social media-based COVID-19 sentiment classification model using Bi-LSTM
**7**	[58]	COVID-19, a tale of two pandemics: Novel coronavirus and fake news messaging
**8**	[59]	Fentanyl panic goes viral: The spread of misinformation about overdose risk from casual contact with fentanyl in mainstream and social media
**9**	[60]	Precision Global Health ‐ The case of Ebola: A scoping review
**10**	[61]	Social Media, Science, and Attack Discourse: How Twitter Discussions of Climate Change Use Sarcasm and Incivility
**11**	[62]	2019-nCoV, fake news, and racism
**12**	[63]	Digital work engagement among Italian neurologists
**13**	[64]	Quantifying the drivers behind collective attention in information ecosystems
**14**	[65]	The Politicization of Ivermectin Tweets during the COVID-19 Pandemic
**15**	[66]	COVID-19 in South Carolina: Experiences Using Facebook as a Self-Organizing Tool for Grassroots Advocacy, Education, and Social Support
**16**	[67]	Self-medication and the ‘infodemic’ during mandatory preventive isolation due to the COVID-19 pandemic
**17**	[68]	How essential is kratom availability and use during COVID-19? Use pattern analysis based on survey and social media data
**18**	[69]	OCR post-correction for detecting adversarial text images
**19**	[70]	INDOBERT FOR INDONESIAN FAKE NEWS DETECTION
**20**	[71]	An entropy-based method to control COVID-19 rumors in online social networks using opinion leaders
**21**	[72]	A systematic literature review on spam content detection and classification
**22**	[73]	How do Canadian public health agencies respond to the COVID-19 emergency using social media: A protocol for a case study using content and sentiment analysis
**23**	[74]	A retrospective analysis of social media posts pertaining to COVID-19 vaccination side effects
**24**	[75]	Quality of Bladder Cancer Information on YouTube[Formula presented]
**25**	[76]	A Relationship-Centered and Culturally Informed Approach to Studying Misinformation on COVID-19
**26**	[77]	It Takes a Village to Combat a Fake News Army: Wikipedia’s Community and Policies for Information Literacy
**27**	[78]	Identifying cross-platform user relationships in 2020 U.S. election fraud and protest discussions
**28**	[79]	Social research 2.0: Virtual snowball sampling method using Facebook
**29**	[80]	Realfood and Cancer: Analysis of the Reliability and Quality of YouTube Content
**30**	[81]	A comprehensive Benchmark for fake news detection
**31**	[82]	A scoping review of COVID-19 online mis/disinformation in Black communities
**32**	[83]	Improving the Communication and Credibility of Government Media in Response to Public Health Emergencies: Analysis of Tweets From the WeChat Official Accounts of 10 Chinese Health Commissioners
**33**	[84]	Light weight recommendation system for social networking analysis using a hybrid BERT-SVM classifier algorithm
**34**	[85]	Fake Sentence Detection Based on Transfer Learning: Applying to Korean COVID‐19 Fake News
**35**	[86]	Social Bots and the Spread of Disinformation in Social Media: The Challenges of Artificial Intelligence
**36**	[87]	Connectivity Between Russian Information Sources and Extremist Communities Across Social Media Platforms
**37**	[88]	Nigeria EndSARS Protest: False Information Mitigation Hybrid Model
**38**	[89]	Arabic Language Modeling Based on Supervised Machine Learning
**39**	[90]	When Does an Individual Accept Misinformation? An Extended Investigation Through Cognitive Modeling
**40**	[91]	COVID-19 and Vitamin D Misinformation on YouTube: Content Analysis
**41**	[92]	Perceived Vaccine Efficacy, Willingness to Pay for COVID-19 Vaccine and Associated Determinants among Foreign Migrants in China
**42**	[93]	Misinformation about the Human Gut Microbiome in YouTube Videos: Cross-sectional Study
**43**	[94]	A Social Network Analysis of Tweets Related to Mandatory COVID-19 Vaccination in Poland
**44**	[95]	MeVer NetworkX: Network Analysis and Visualization for Tracing Disinformation
**45**	[96]	Fine-Tuning BERT Models to Classify Misinformation on Garlic and COVID-19 on Twitter
**46**	[97]	‘Blurred boundaries’: When nurses and midwives give anti-vaccination advice on Facebook
**47**	[98]	PM Me the Truth? The Conditional Effectiveness of Fact-Checks Across Social Media Sites
**48**	[99]	Xenophobic Bullying and COVID-19: An Exploration Using Big Data and Qualitative Analysis
**49**	[100]	BreadTube Rising: How Modern Creators Use Cultural Formats to Spread Countercultural Ideology
**50**	[101]	Dynamic Light Weight Recommendation System for Social Networking Analysis Using a Hybrid LSTM-SVM Classifier Algorithm
**51**	[102]	An Explainable Fake News Detector Based on Named Entity Recognition and Stance Classification Applied to COVID-19
**52**	[103]	Understanding Public Perceptions of Per- and Polyfluoroalkyl Substances: Infodemiology Study of Social Media
**53**	[104]	Discussions of Asperger Syndrome on Social Media: Content and Sentiment Analysis on Twitter
**54**	[105]	Public Policy Measures to Increase Anti-SARS-CoV-2 Vaccination Rate in Russia
**55**	[106]	Contextualizing Engagement With Health Information on Facebook: Using the Social Media Content and Context Elicitation Method
**56**	[107]	The Challenge of Debunking Health Misinformation in Dynamic Social Media Conversations: Online Randomized Study of Public Masking During COVID-19
**57**	[108]	People lie, actions Don’t! Modeling infodemic proliferation predictors among social media users
**58**	[109]	CB-Fake: A multimodal deep learning framework for automatic fake news detection using capsule neural network and BERT
**59**	[110]	Evaluating the Influence of Twitter Bots via Agent-Based Social Simulation
**60**	[111]	Receiving COVID-19 Messages on Social Media to the People of Semarang City
**61**	[112]	Impact of COVID-19 on HIV Prevention Access: A Multi-platform Social Media Infodemiology Study
**62**	[113]	Monkeypox Vaccine Acceptance among Ghanaians: A Call for Action
**63**	[114]	Conspiracy Beliefs, Misinformation, Social Media Platforms, and Protest Participation
**64**	[115]	State vs. anti-vaxxers: Analysis of Covid-19 echo chambers in Serbia
**65**	[116]	Fake News Detection Techniques on Social Media: A Survey
**66**	[117]	Inclusive Study of Fake News Detection for COVID-19 with New Dataset using Supervised Learning Algorithms
**67**	[118]	On Politics and Pandemic: How Do Chilean Media Talk about Disinformation and Fake News in Their Social Networks?
**68**	[119]	COMMENT: Narrative-based misinformation in India about protection against Covid-19: Not just another "moo-point"
**69**	[120]	Narratives of Anti‐Vaccination Movements in the German and Brazilian Twittersphere: A Grounded Theory Approach
**70**	[121]	Sentiment Analysis on COVID-19 Twitter Data Streams Using Deep Belief Neural Networks
**71**	[122]	Looking for cystoscopy on YouTube: Are videos a reliable information tool for internet users?
**72**	[123]	A Taxonomy of Fake News Classification Techniques: Survey and Implementation Aspects
**73**	[124]	The Impact of the COVID-19 “Infodemic” on Well-Being: A Cross-Sectional Study
**74**	[125]	Medical and Health-Related Misinformation on Social Media: Bibliometric Study of the Scientific Literature
**75**	[126]	Dynamics of social corrections to peers sharing COVID-19 misinformation on WhatsApp in Brazil
**76**	[127]	A hierarchical network-oriented analysis of user participation in misinformation spread on WhatsApp
**77**	[128]	Tweeting on COVID-19 pandemic in South Africa: LDA-based topic modelling approach
**78**	[129]	Factors Influencing the Accessibility and Reliability of Health Information in the Face of the COVID-19 Outbreak—A Study in Rural China
**79**	[130]	The Plebeian Algorithm: A Democratic Approach to Censorship and Moderation
**80**	[131]	Tracking Private WhatsApp Discourse about COVID-19 in Singapore: Longitudinal Infodemiology Study
**81**	[132]	The Impact of COVID-19 on Conspiracy Hypotheses and Risk Perception in Italy: Infodemiological Survey Study Using Google Trends
**82**	[133]	What and Why? Towards Duo Explainable Fauxtography Detection Under Constrained Supervision
**83**	[134]	Public perception of SARS-CoV-2 vaccinations on social media: Questionnaire and sentiment analysis
**84**	[135]	Identifying Covid-19 misinformation tweets and learning their spatio-temporal topic dynamics using Nonnegative Coupled Matrix Tensor Factorization
**85**	[136]	Cultural Evolution and Digital Media: Diffusion of Fake News About COVID-19 on Twitter
**86**	[137]	Covid-19 vaccine hesitancy on social media: Building a public twitter data set of antivaccine content, vaccine misinformation, and conspiracies
**87**	[138]	News media stories about cancer on Facebook: How does story framing influence response framing, tone and attributions of responsibility?
**88**	[139]	Credibility of scientific information on social media: Variation by platform, genre and presence of formal credibility cues
**89**	[140]	Health Misinformation on Social Media and its Impact on COVID-19 Vaccine Inoculation in Jordan
**90**	[141]	Infodemia–an analysis of fake news in polish news portals and traditional media during the coronavirus pandemic
**91**	[142]	Feasibility of utilizing social media to promote hpv self‐collected sampling among medically underserved women in a rural southern city in the united states (U.s.)
**92**	[143]	A retrospective analysis of the covid-19 infodemic in Saudi Arabia
**93**	[144]	Machine learning in detecting covid-19 misinformation on twitter
**94**	[145]	The Response of Governments and Public Health Agencies to COVID-19 Pandemics on Social Media: A Multi-Country Analysis of Twitter Discourse
**95**	[146]	Human Papillomavirus Vaccination and Social Media: Results in a Trial With Mothers of Daughters Aged 14–17
**96**	[147]	Social media monitoring of the COVID-19 pandemic and influenza epidemic with adaptation for informal language in Arabic twitter data: Qualitative study
**97**	[148]	An infodemiology and infoveillance study on covid-19: Analysis of twitter and google trends
**98**	[149]	COVIDSenti: A Large-Scale Benchmark Twitter Data Set for COVID-19 Sentiment Analysis
**99**	[150]	A survey of Big Data dimensions vs Social Networks analysis
**100**	[151]	Plandemic Revisited: A Product of Planned Disinformation Amplifying the COVID-19 “infodemic”
**101**	[152]	Marginalizing the Mainstream: How Social Media Privilege Political Information
**102**	[153]	QATAR’S COMMUNICATION STRATEGY AND THE RESOLUTION OF THE DIPLOMATIC CONFLICT IN THE GULF
**103**	[154]	Towards a critical understanding of social networks for the feminist movement: Twitter and the women’s strike
**104**	[155]	YouTube as a source of information on gout: a quality analysis
**105**	[156]	Social Media, Cognitive Reflection, and Conspiracy Beliefs
**106**	[157]	Using machine learning to compare provaccine and antivaccine discourse among the public on social media: Algorithm development study
**107**	[158]	A social bot in support of crisis communication: 10-years of @LastQuake experience on Twitter
**108**	[159]	Determinants of individuals’ belief in fake news: A scoping review determinants of belief in fake news
**109**	[160]	Lack of trust, conspiracy beliefs, and social media use predict COVID-19 vaccine hesitancy
**110**	[161]	Health information seeking behaviors on social media during the covid-19 pandemic among american social networking site users: Survey study
**111**	[162]	Semi-automatic generation of multilingual datasets for stance detection in Twitter
**112**	[163]	Social media content of idiopathic pulmonary fibrosis groups and pages on facebook: Cross-sectional analysis
**113**	[164]	Collecting a large scale dataset for classifying fake news tweets usingweak supervision
**114**	[165]	Youtube videos and informed decision-making about covid-19 vaccination: Successive sampling study
**115**	[166]	The commonly utilized natural products during the COVID-19 pandemic in Saudi Arabia: A cross-sectional online survey
**116**	[167]	A behavioural analysis of credulous Twitter users
**117**	[73]	How do Canadian public health agencies respond to the COVID-19 emergency using social media: A protocol for a case study using content and sentiment analysis
**118**	[168]	The negative role of social media during the COVID-19 outbreak
**119**	[169]	Twitter’s Role in Combating the Magnetic Vaccine Conspiracy Theory: Social Network Analysis of Tweets
**120**	[58]	COVID-19, a tale of two pandemics: Novel coronavirus and fake news messaging
**121**	[170]	Concerns discussed on chinese and french social media during the COVID-19 lockdown:comparative infodemiology study based on topic modeling
**122**	[171]	Social media and medical education in the context of the COVID-19 pandemic: Scoping review
**123**	[172]	Rumor Detection Based on SAGNN: Simplified Aggregation Graph Neural Networks
**124**	[173]	Detecting fake news on Facebook: The role of emotional intelligence
**125**	[174]	Information disorders during the COVID-19 infodemic: The case of Italian Facebook
**126**	[175]	Conspiracy vs science: A large-scale analysis of online discussion cascades
**127**	[176]	Will the World Ever Be the Same After COVID-19? Two Lessons from the First Global Crisis of a Digital Age
**128**	[177]	Using tweets to understand how COVID-19–Related health beliefs are affected in the age of social media: Twitter data analysis study
**129**	[178]	General audience engagement with antismoking public health messages across multiple social media sites: Comparative analysis
**130**	[179]	An analysis of YouTube videos as educational resources for dental practitioners to prevent the spread of COVID-19
**131**	[180]	Detection of Fake News Text Classification on COVID-19 Using Deep Learning Approaches
**132**	[181]	Visual analytics of twitter and social media dataflows: A casestudy of COVID-19 rumors
**133**	[182]	Examining embedded apparatuses of AI in Facebook and TikTok
**134**	[183]	Prevalence and perception among saudi arabian population about resharing of information on social media regarding natural remedies as protective measures against covid-19
**135**	[184]	Level of acceptance of news stories on social media platforms among youth in Nigeria
**136**	[185]	Disinformation, vaccines, and covid-19. Analysis of the infodemic and the digital conversation on twitter [Desinformación, vacunas y covid-19. Análisis de la infodemia y la conversación digital en twitter]
**137**	[186]	Development and testing of a multi-lingual Natural Language Processing-based deep learning system in 10 languages for COVID-19 pandemic crisis: A multi-center study
**138**	[187]	Youtube as a source of information on epidural steroid injection
**139**	[188]	An exploratory study of social media users’ engagement with COVID-19 vaccine-related content
**140**	[189]	Online influencers: Healthy food or fake news
**141**	[190]	Sentimental Analysis on Health-Related Information with Improving Model Performance using Machine Learning
**142**	[191]	Digital civic participation and misinformation during the 2020 taiwanese presidential election
**143**	[192]	Challenging post-communication: Beyond focus on a ‘few bad apples’ to multi-level public communication reform
**144**	[193]	Knowledge about COVID-19 in Brazil: Cross-sectional web-based study
**145**	[194]	“Down the rabbit hole” of vaccine misinformation on youtube: Network exposure study
**146**	[195]	Exploring Adversarial Attacks and Defences for Fake Twitter Account Detection
**147**	[196]	Social Media Use by Young People Living in Conflict-Affected Regions of Myanmar
**148**	[197]	Two-Path Deep Semisupervised Learning for Timely Fake News Detection
**149**	[198]	Deep learning for misinformation detection on online social networks: a survey and new perspectives
**150**	[199]	FauxWard: a graph neural network approach to fauxtography detection using social media comments
**151**	[200]	Internet users engage more with phatic posts than with health misinformation on Facebook
**152**	[201]	SENTIMENTAL ANALYSIS OF COVID-19 TWITTER DATA USING DEEP LEARNING AND MACHINE LEARNING MODELS [ANÁLISIS DE SENTIMIENTO DE LOS DATOS DE TWITTER DE COVID-19 UTILIZANDO MODELOS DE APRENDIZAJE PROFUNDO Y APRENDIZAJE MÁQUINA]
**153**	[202]	Partisan public health: how does political ideology influence support for COVID-19 related misinformation?
**154**	[203]	COVID-19 and the “Film Your Hospital” conspiracy theory: Social network analysis of Twitter data
**155**	[204]	Fake news and aggregated credibility: Conceptualizing a co-creative medium for evaluation of sources online
**156**	[205]	COVID-19 Information on YouTube: Analysis of Quality and Reliability of Videos in Eleven Widely Spoken Languages across Africa
**157**	[206]	COVID-19: Retransmission of official communications in an emerging pandemic
**158**	[207]	Insights from twitter conversations on lupus and reproductive health: Protocol for a content analysis
**159**	[208]	Temporal and location variations, and link categories for the dissemination of COVID-19-related information on twitter during the SARS-CoV-2 outbreak in Europe: Infoveillance study
**160**	[209]	Inflaming public debate: a methodology to determine origin and characteristics of hate speech about sexual and gender diversity on Twitter
**161**	[210]	How to fight an infodemic: The four pillars of infodemic management
**162**	[211]	Genesis of an emergency public drug information website by the French Society of Pharmacology and Therapeutics during the COVID-19 pandemic
**163**	[212]	YouTube as a source of information on COVID-19: A pandemic of misinformation?
**164**	[213]	The impact of social media on panic during the COVID-19 pandemic in iraqi kurdistan: Online questionnaire study
**165**	[214]	COVID-19 and the 5G conspiracy theory: Social network analysis of twitter data
**166**	[215]	From disinformation to fact-checking: How Ibero-American fact-checkers on Twitter combat fake news
**167**	[216]	Tracking social media discourse about the COVID-19 pandemic: Development of a public coronavirus Twitter data set
**168**	[217]	Mining physicians’ opinions on social media to obtain insights into COVID-19: Mixed methods analysis
**169**	[218]	A new application of social impact in social media for overcoming fake news in health
**170**	[219]	Islamophobic hate speech on social networks. An analysis of attitudes to Islamophobia on Twitter [El discurso de odio islamófobo en las redes sociales. Un análisis de las actitudes ante la islamofobia en Twitter]
**171**	[220]	Information management in healthcare and environment: Towards an automatic system for fake news detection
**172**	[221]	Vaccine-related advertising in the Facebook Ad Archive
**173**	[222]	Ontology Meter for Twitter Fake Accounts Detection
**174**	[223]	Social media and fake news in the post-truth era: The manipulation of politics in the election process
**175**	[224]	An analysis of fake narratives on social media during 2019 Indonesian presidential election
**176**	[225]	Unlink the link between COVID-19 and 5G Networks: an NLP and SNA based Approach
**177**	[226]	Fake News Detection Using Machine Learning Ensemble Methods
**178**	[227]	Social Network Analysis of COVID-19 Public Discourse on Twitter: Implications for Risk Communication
**179**	[228]	Lies Kill, Facts Save: Detecting COVID-19 Misinformation in Twitter
**180**	[229]	The visual vaccine debate on twitter: A social network analysis
**181**	[230]	"Tell us what’s going on": Exploring the information needs of pregnant and postpartum women in Australia during the pandemic with ’Tweets’, ’Threads’, and women’s views
**182**	[231]	Paying SPECIAL consideration to the digital sharing of information during the COVID-19 pandemic and beyond
**183**	[232]	Multiple social platforms reveal actionable signals for software vulnerability awareness: A study of GitHub, Twitter and Reddit
**184**	[233]	Fake news analysis modeling using quote retweet
**185**	[234]	Automatically appraising the credibility of vaccine-related web pages shared on social media: A twitter surveillance study
**186**	[235]	Citizen journalism and public participation in the Era of New Media in Indonesia: From street to tweet
**187**	[236]	Disinformation and vaccines on social networks: Behavior of hoaxes on Twitter [Desinformación y vacunas en redes: Comportamiento de los bulos en Twitter]
**188**	[237]	Fiji’s coup culture: Rediscovering a voice at the ballot box
**189**	[238]	Polarization and fake news: Early warning of potential misinformation targets
**190**	[239]	Fake news and dental education
**191**	[240]	A corpus of debunked and verified user-generated videos
**192**	[241]	Comparison study between the UAE, the UK, and India in Dealing with whatsapp fake news
**193**	[242]	Constitution, democracy, regulation of the internet and electoral fake news in the Brazilian elections [Constituição, democracia, regulação da internet e fake news nas eleições brasileiras]
**194**	[243]	Recycling old strategies and devices: What remains, an art project addressing disinformation campaigns (Re)using strategies to delay industry regulation [What remains, un proyecto artístico que trata sobre las campañas de desinformación (Re)utilizando estrategias para retrasar la regulación industrial]
**195**	[244]	Reading between the lines and the numbers: An analysis of the first NetzDG reports
**196**	[245]	After the ‘APIcalypse’: social media platforms and their fight against critical scholarly research
**197**	[246]	Health-Related Disaster Communication and Social Media: Mixed-Method Systematic Review
**198**	[247]	Are internet videos useful sources of information during global public health emergencies? A case study of YouTube videos during the 2015–16 Zika virus pandemic
**199**	[248]	Causal language and strength of inference in academic and media articles shared in social media (CLAIMS): A systematic review
**200**	[249]	Detection and visualization of misleading content on Twitter
**201**	[250]	Tweet, truth and fake news: A study of BJP’s official tweeter handle
**202**	[251]	Social media, dietetic practice and misinformation: A triangulation research
**203**	[252]	Examination of YouTube videos related to synthetic cannabinoids
**204**	[253]	Practices and promises of Facebook for science outreach: Becoming a “Nerd of Trust”
**205**	[254]	Rising tides or rising stars?: Dynamics of shared attention on twitter during media events
**206**	[255]	Misleading health-related information promoted through video-based social media: Anorexia on youtube
**207**	[256]	Quality of healthcare information on YouTube: psoriatic arthritis [Qualität von Gesundheitsinformationen auf YouTube: Psoriasisarthritis]
**208**	[257]	YOUTUBEASASOURCE OFINFORMATIONABOUT UNPROVENDRUGSFOR COVID-19: the role of the mainstream media and recommendation algorithms in promoting misinformation [YOUTUBE COMO FUENTE DE INFORMACIÓN SOBRE MEDICAMENTOS NO PROBADOS PARA EL COVID-19: el papel de los principales medios de comunicación y los algoritmos de recomendación en la promoción de la desinformación] [YOUTUBE COMO FONTE DE INFORMAÇÃO SOBRE MEDICAMENTOS SEM EFICÁCIA COMPROVADA PARA COVID-19: o papel da imprensa tradicional e dos algoritmos de recomendação na promoção da desinformação]
**209**	[258]	Utilising online eye-tracking to discern the impacts of cultural backgrounds on fake and real news decision-making
**210**	[259]	Top 100 #PCOS influencers: Understanding who, why and how online content for PCOS is influenced
**211**	[260]	Twitter Trends for Celiac Disease and the Gluten-Free Diet: Cross-sectional Descriptive Analysis
**212**	[261]	Negative COVID-19 Vaccine Information on Twitter: Content Analysis
**213**	[262]	Platform Effects on Public Health Communication:A Comparative and National Study of Message Design and Audience Engagement Across Twitter and Facebook
**214**	[263]	The influence of fake news on face-trait learning
**215**	[264]	COVID-Related Misinformation Migration to BitChute and Odysee
**216**	[265]	Sending News Back Home: Misinformation Lost in Transnational Social Networks
**217**	[266]	Public Opinion Manipulation on Social Media: Social Network Analysis of Twitter Bots during the COVID-19 Pandemic
**218**	[267]	Organization and evolution of the UK far-right network on Telegram
**219**	[268]	Predictive modeling for suspicious content identification on Twitter
**220**	[269]	Detection and moderation of detrimental content on social media platforms: current status and future directions
**221**	[270]	Cross-platform information spread during the January 6th capitol riots
**222**	[271]	Combating multimodal fake news on social media: methods, datasets, and future perspective
**223**	[272]	In.To. COVID-19 socio-epidemiological co-causality
**224**	[273]	Cross-platform analysis of public responses to the 2019 Ridgecrest earthquake sequence on Twitter and Reddit
**225**	[274]	Investigating the Impacts of YouTube’s Content Policies on Journalism and Political Discourse
**226**	[275]	Fake or real news about COVID-19? Pretrained transformer model to detect potential misleading news
**227**	[276]	A deep dive into COVID-19-related messages on WhatsApp in Pakistan
**228**	[277]	It-which-must-not-be-named: COVID-19 misinformation, tactics to profit from it and to evade content moderation on YouTube
**229**	[278]	Understanding the Social Mechanism of Cancer Misinformation Spread on YouTube and Lessons Learned: Infodemiological Study
**230**	[279]	The three-step persuasion model on YouTube: A grounded theory study on persuasion in the protein supplements industry
**231**	[280]	Examining the Twitter Discourse on Dementia During Alzheimer’s Awareness Month in Canada: Infodemiology Study
**232**	[281]	Rapid Sharing of Islamophobic Hate on Facebook: The Case of the Tablighi Jamaat Controversy
**233**	[282]	Social Media and the Influence of Fake News on Global Health Interventions: Implications for a Study on Dengue in Brazil
**234**	[283]	Spanish Facebook Posts as an Indicator of COVID-19 Vaccine Hesitancy in Texas
**235**	[284]	Fine-tuned Sentiment Analysis of COVID-19 Vaccine-Related Social Media Data: Comparative Study
**236**	[285]	Empowering Health Care Workers on Social Media to Bolster Trust in Science and Vaccination During the Pandemic: Making IMPACT Using a Place-Based Approach
**237**	[286]	Exploring Motivations for COVID-19 Vaccination among Black Young Adults in 3 Southern US States: Cross-sectional Study
**238**	[287]	Development of Principles for Health-Related Information on Social Media: Delphi Study
**239**	[288]	The Influence of Fake News on Social Media: Analysis and Verification of Web Content during the COVID-19 Pandemic by Advanced Machine Learning Methods and Natural Language Processing
**240**	[289]	Habermasian analysis of reports on Presidential tweets influencing politics in the USA
**241**	[290]	A unified approach of detecting misleading images via tracing its instances on web and analyzing its past context for the verification of multimedia content
**242**	[291]	“It’s true! I saw it on WhatsApp”: Social Media, Covid-19, and Political-Ideological Orientation in Brazil
**243**	[292]	Use of digital media for family planning information by women and their social networks in Kenya: A qualitative study in peri-urban Nairobi
**244**	[293]	Search Term Identification Methods for Computational Health Communication: Word Embedding and Network Approach for Health Content on YouTube
**245**	[294]	Bots’ Activity on COVID-19 Pro and Anti-Vaccination Networks: Analysis of Spanish-Written Messages on Twitter
**246**	[295]	Misinformation About COVID-19 Vaccines on Social Media: Rapid Review
**247**	[296]	Fear, Stigma and Othering: The Impact of COVID-19 Rumours on Returnee Migrants and Muslim Populations of Nepal
**248**	[297]	Tackling fake news in socially mediated public spheres: A comparison of Weibo and WeChat
**249**	[298]	The Networked Context of COVID-19 Misinformation: Informational Homogeneity on YouTube at the Beginning of the Pandemic
**250**	[299]	Twelve tips to make successful medical infographics
**251**	[300]	TClustVID: A novel machine learning classification model to investigate topics and sentiment in COVID-19 tweets
**252**	[301]	Cognitive and affective responses to political disinformation in Facebook
**253**	[302]	Experience: Managing misinformation in social media-insights for policymakers from Twitter analytics
**254**	[303]	Hepatitis E vaccine in China: Public health professional perspectives on vaccine promotion and strategies for control
**255**	[304]	“Fake Elections”? Cyber Propaganda, Disinformation and the 2017 General Elections in Kenya
**256**	[305]	‘Fake News’ in urology: evaluating the accuracy of articles shared on social media in genitourinary malignancies
**257**	[306]	“I will kill myself”–The series of posts in Facebook and unnoticed departure of a life
**258**	[307]	Ethiopia’s Hate Speech Predicament: Seeking Antidotes Beyond a Legislative Response
**259**	[308]	The Paradox of Participation Versus Misinformation: Social Media, Political Engagement, and the Spread of Misinformation
**260**	[309]	‘Techlash’, responsible innovation, and the self-regulatory organization
**261**	[310]	YouTube videos as a source of misinformation on idiopathic pulmonary fibrosis
**262**	[311]	Dissemination of Misinformative and Biased Information about Prostate Cancer on YouTube
**263**	[312]	Hyperacusis and social media trends
**264**	[313]	Media education with the monetization of YouTube: The loss of truth as an exchange value [Educación mediática frente a la monetización en YouTube: la pérdida de la verdad como valor de cambio]
**265**	[314]	All i Have Learned, i Have Learned from Google: Why Today’s Facial Rejuvenation Patients are Prone to Misinformation, and the Steps We can take to Contend with Unreliable Information
**266**	[315]	Digital diplomacy: Emotion and identity in the public realm
**267**	[316]	Drug information, misinformation, and disinformation on social media: a content analysis study
**268**	[317]	Mining significant microblogs for misinformation identification: An attention-based approach
**269**	[318]	The web and public confidence in MMR vaccination in Italy
**270**	[319]	Using Twitter to communicate conservation science from a professional conference
**271**	[320]	Communication in the face of a school crisis: Examining the volume and content of social media mentions during active shooter incidents
**272**	[321]	Media and public reactions toward vaccination during the ’hepatitis B vaccine crisis’ in China
**273**	[322]	#FluxFlow: Visual analysis of anomalous information spreading on social media
**274**	[323]	Social media in health ‐ what are the safety concerns for health consumers?
**275**	[324]	Internet and electronic resources for inflammatory bowel disease: A primer for providers and patients
**276**	[325]	Fukushima, Facebook and Feeds: Informing the Public in a Digital Era
**277**	[326]	A graph-theoretic embedding-based approach for rumor detection in twitter
**278**	[327]	Investigating Facebook’s interventions against accounts that repeatedly share misinformation
**279**	[328]	Can technological advancements help to alleviate COVID-19 pandemic? a review
**280**	[126]	Dynamics of social corrections to peers sharing COVID-19 misinformation on WhatsApp in Brazil
**281**	[329]	Antibiotics for acne vulgaris: using instagram to seek insight into the patient perspective
**282**	[330]	Pre-emption strategies to block taxes on sugar-sweetened beverages: A framing analysis of Facebook advertising in support of Washington state initiative-1634
**283**	[331]	COVID-19: fighting panic with information
**284**	[332]	Going beyond fact-checking to fight health misinformation: A multi-level analysis of the Twitter response to health news stories

### Documents versus social networks

**Fig 11:**
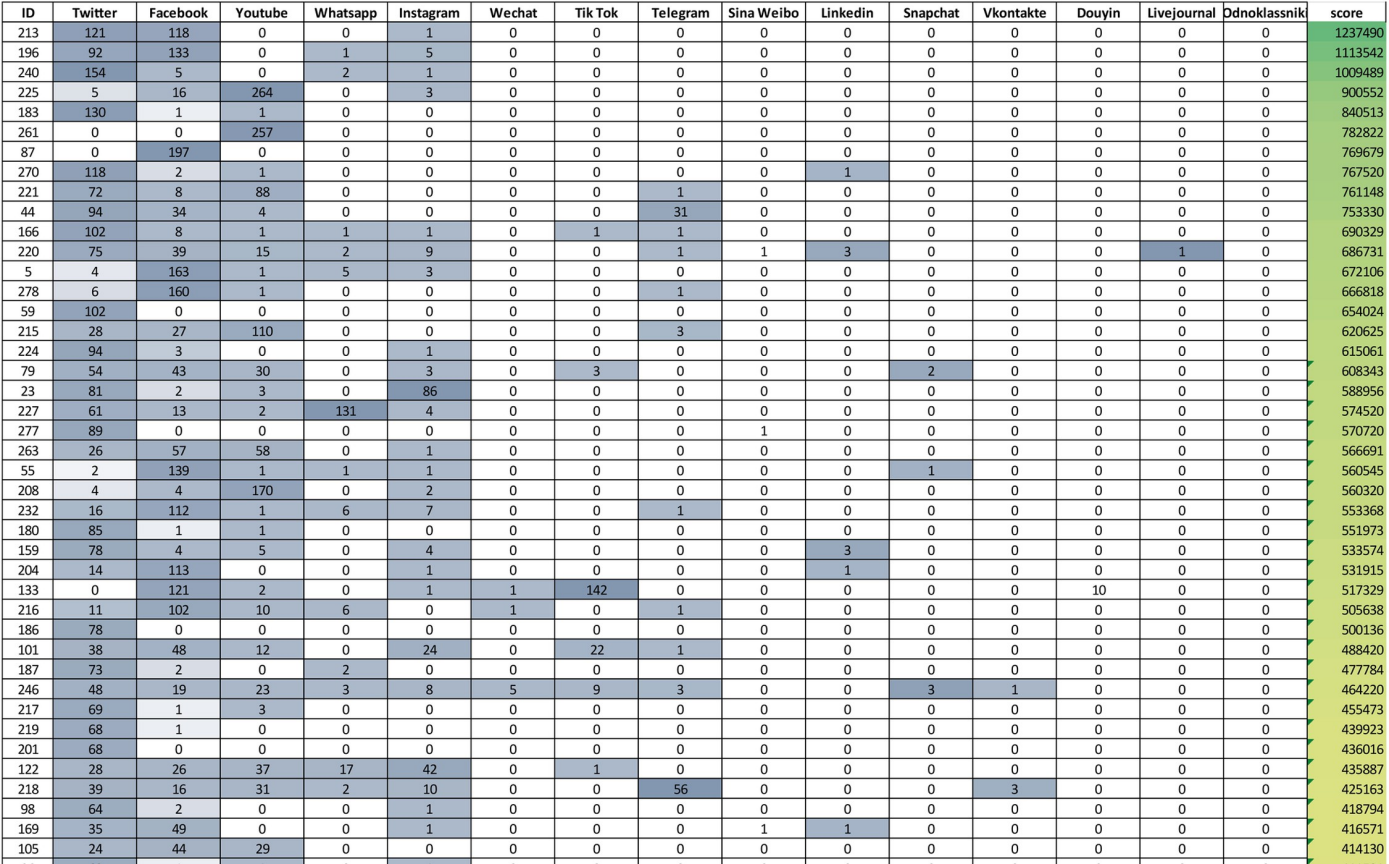
Excerpt of the documents-social networks matrix. The matrix (size 283X15) reports the strength of relationships between documents and social media platforms.

The number of social media platforms included in the initial query on the Scopus database has been deemed to be enough complete in terms of customers’ propensity. We have assumed that other scholars have addressed a certain topic and some social network platforms relevant for that topic in the same article. Moreover, we also assumed that the frequency of usage of a social media name (e.g. Facebook) within the document text is a meaningful proxy measure of the relevance of the correspondent social media platform for that particular paper. Starting from these considerations, fifteen nodes have been added to the Obsidian vault, one per each social media platform present in the corpus, since neither “Moir Mir” nor “Kuaishou” platforms are not present. The relationships between documents and social media can be found in the graph topology, as they become the ties between documents nodes and social media nodes, in principle allowing to evaluate the importance of the social media through the corpus. In Fig 11 the number in the cells represent the number of links connecting a document (row) with a social media platform name (column).

For each social media the greyscale intensity gives visual feedback (the darker, the highest) upon the number of outgoing links from the document with it. The total number of incoming links for each social network (*Representativity*) can be computed by summing each column separately and represents how much the social network is addressed by the entire corpus (Twitter is the most represented platform in the corpus, the rightmost–Odnoklassniki–is the less represented). The documents also have been sorted on the *Ranking score*, that is the sum of the outgoing links scaled by the Representativity of the social media, reported on the greenish rightmost column.

### Documents versus topics

The nodes corresponding to the topics have been constructed associatively from bag-of-words and clusters obtained in the previous phases. It is necessary to first detect the corresponding tags and then observe how they are highlighted in the various notes/documents. Clusters have been formed and then identified as topics according to the customary process of topic modeling. The identified topics are: *Politics* (addressing political events and issues); *Health and Science* (mainly regarding Covid-19 outbreak, debates about vaccines and drugs, but also environmental pollution and climate change, as well as technological and scientific development); *Social Issues* (Current social issues, such as immigration, wars, gender issues, poverty, and racism); *Disasters and Tragedies* (Criminal events, massacres, terrorist attacks, and natural disasters that have polarized social media users); *Economy and Finance* (topics related to the performance of financial markets, cryptocurrencies, investors, and relationships with various stakeholders); *Other* (Cluster gathering minor topics not falling under the previous ones, such as gossip about celebrities, unclassifiable conspiracy theories, internet memes, and generic hoaxes).

**Fig 12:**
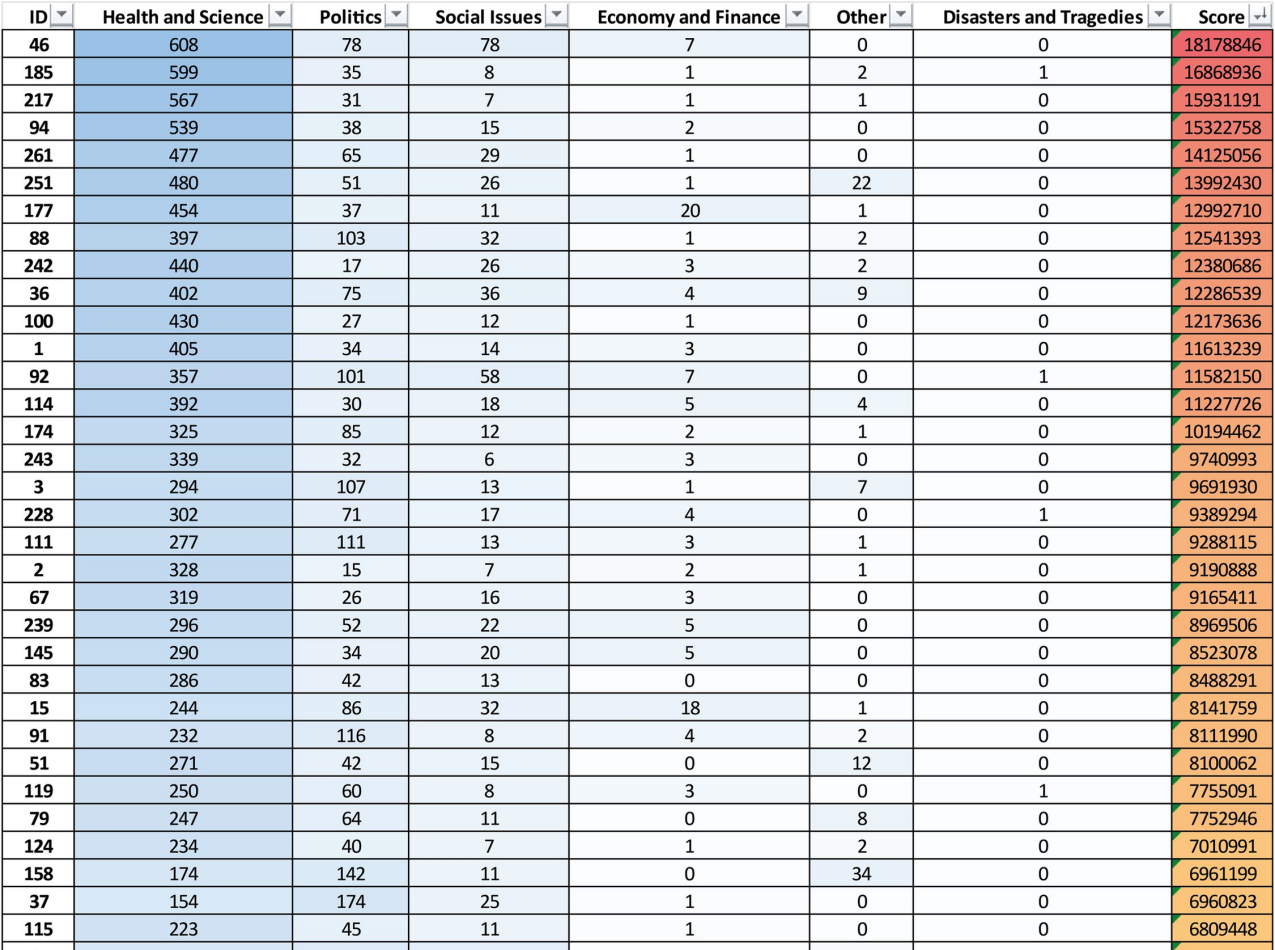
Excerpt of the documents-topics matrix. The matrix (size 283X6) reports the strength of relationships between documents and topics.

There are considerable differences in the amount of ID addressed per specific topic in the 283 articles analyzed. In Fig 12 the values in the cells represent the number of links connecting a document (row) with a topic (column).

For each social media the blue intensity gives visual feedback (the darker, the highest) upon the number of outgoing links from the document to the social media. The total number of incoming links (*Relevance*) can be computed for each topic by summing the values along each column, it represents how much the topic is addressed by the entire corpus. From column sorting, it is evident that Health and Science is the most represented topic in the corpus, while the rightmost–Disasters and Tragedies–is the less represented,

The documents also have been sorted on the *Ranking score*, that is the sum of the outgoing links scaled by the Relevance of the topics, reported on the reddish rightmost column.

Once assessed the overall Relevance of the topics over the entire documental corpus, it is possible to rank them from the most relevant to the lowest in terms of total links to documents: *Health and Science* (27197 links), *Politics* (15082), *Social Issues* (5885), *Economy and Finance* (1092), *Other* (663), *Disasters and Tragedies* (565). *Health and Science*, *Politics*, *Social Issues* are the most relevant, which is consistent with the analysis performed in the early stage of the primary sources’ selection of the articles (§ 2.1).

From the bag-of-words is clear that the relevance of *Health and Science* is mainly due to the recent global pandemic that has been the subject of both correct and false information. The rush to find vaccines to tackle the Covid-19 pandemic ignited a flamed discourse on big pharma companies on which many conspiracy theories thrived. The phenomenon, however, is confused with the search for alternative information to traditional sources [332].

ID on *Politics* topic mainly relates to the political events happened after 2016 such as the Brexit, the USA presidential election, the Russia-Gate, the rise of nationalist movements worldwide, the cold conflict between the USA and North Korea, and the actions of dissidents against Vladimir Putin.

The issues regarding migratory phenomena, cultural, religious, sexual autonomy, or gender self-determination are always subject to heated debates among individuals, stirring them up. This instinctive response to topics that touch upon personal spheres and intimate beliefs is often exploited as a mechanism to deactivate critical control over one’s conscious actions. Users of various social media platforms, driven by fervor, tend to share messages with other users, regardless of their positions on the matter. The content sharing mechanism, facilitated by design through the interface of major social networking platforms, is constantly exploited to disseminate ID, as evidenced by the ranking of *Social Issues* in Fig 12.

When it comes to tragedies and natural disasters, however, this sharing mechanism is seldom utilized. Apparently, events that touch people not only from the perspective of beliefs but also through empathetic proximity to their fellow human beings do not spread false information as effectively [333,334].

### Social media platforms versus topics

**Fig 13:**
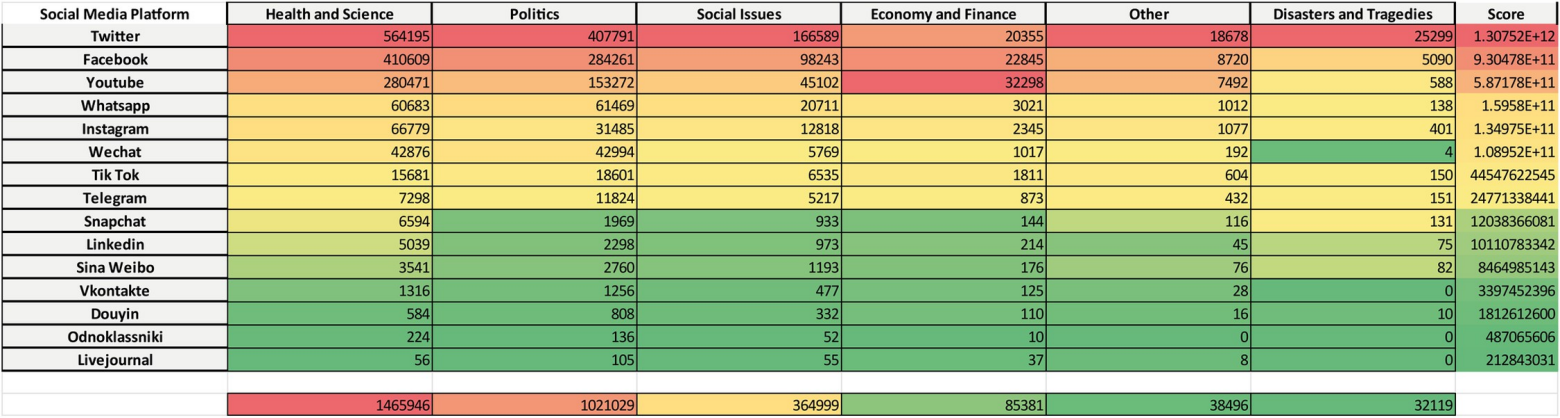
The social media platforms-topics matrix. Each cell reports the corresponding strength of relationship. The bottom row represents the total sums of the strengths per topic. The rightmost column reports a weighted score for each social media platform.

As described earlier, both the connections between documents and social networks, and the connections between documents and topics were obtained as relationships between corresponding nodes within the Obsidian vault. Each of these connections represents a pathway from a document to a topic or a social network. Consequently, it is possible to identify those documents that bridge the gap between topics and social networks and determine the level of connection between these two node types. Following this logic, we have obtained the social network versus topics matrix shown in the Fig 13.

Such relationship can be interpreted as the *Eco* of that topic in the particular *Eco-chamber* represented by the social media corresponding to that row. For example, the Health and Science resonates in descending order in Twitter, Facebook, Youtube, Whatsapp, Instagram, Wechat, and so on, as visually suggested by the color scale: reddish are worst, greenish are better in terms of ID spreading. The overall Eco is reported on the last row. As previously done, the score accounts for the relative importance of the social media platforms as eco-chambers.

**Fig 14:**
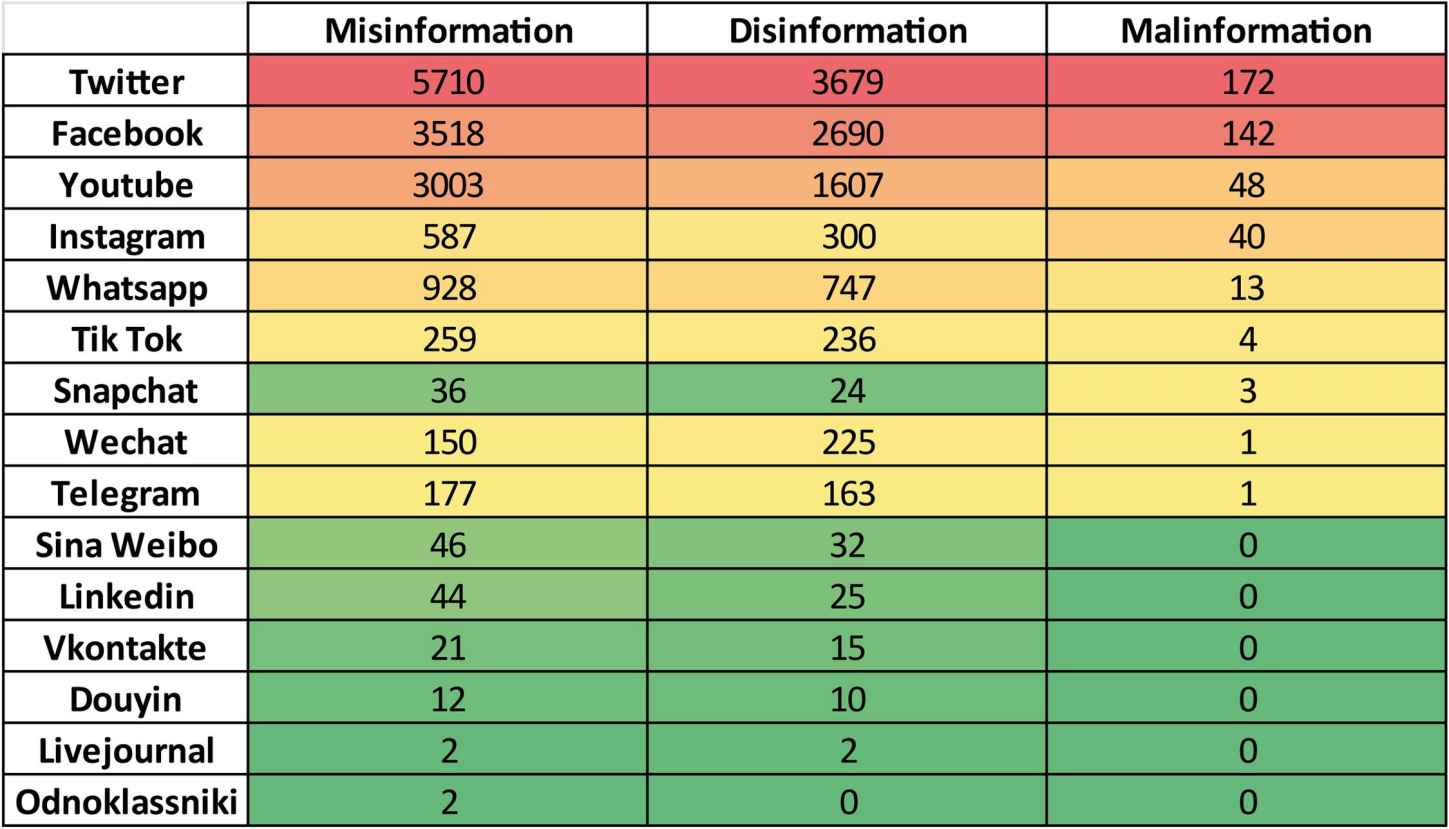
Correlation matrix between ID types. The ID types, misinformation, disinformation, malinformation, are correlated with the most widely used social networks worldwide.

As expected, *reverberation* as an echo-chamber is proportional to the diffusion of the corresponding platform, since this is precisely the mechanism on which its relevance is based: the revenue mechanism on which all social networks are based is precisely the number of subscribers and the possibility of showing advertising content to, or collecting data, from as many people. As a consequence of how they are designed, social networking platforms favor the sharing of content as the basic mechanism for establishing and strengthening social relations between users. This is reflected in both the economic importance of the platform and its ability to *amplify* shared information, whether true or false. Therefore, it is not surprising that the ordering of the score corresponds to the ordering of notoriety of the platform. Actually, the score reflects the economic importance of the platform only partially [335]. This discrepancy could be associated with the sharing mechanism and the type of content on which the platform is based on. Tagging someone else tweet is much easier and faster than watching an entire video on YouTube and posting a comment. From another perspective, spreading ID content is harder on platforms like Tik Tok even though they have a large diffusion, especially among younger generations. Fig 14 shows how different types of IDs move through the major social networks.

Obviously, as noted above, Twitter and Facebook are the social networks where fake news is spread the most. Among the ID types, on the other hand, it is clear how misinformation, i.e., incorrect information disseminated without intent to deceive or harm, is most prevalent. Disinformation (the intentional manipulation of false news), is in second place. The dissemination of distorted news with intent to deceive or harm (malinformation) accounts for a much lower proportion. This result shows that most users are not aware that they are spreading ID. There is thus evidence that there is a strong users’ ingenuity in the sharing of content and that users often share so much for the sake of an exchange of any kind rather than for reasons driven by real critical thinking [336–339].

### The role of AI: Aid or pitfall?

**Fig 15:**
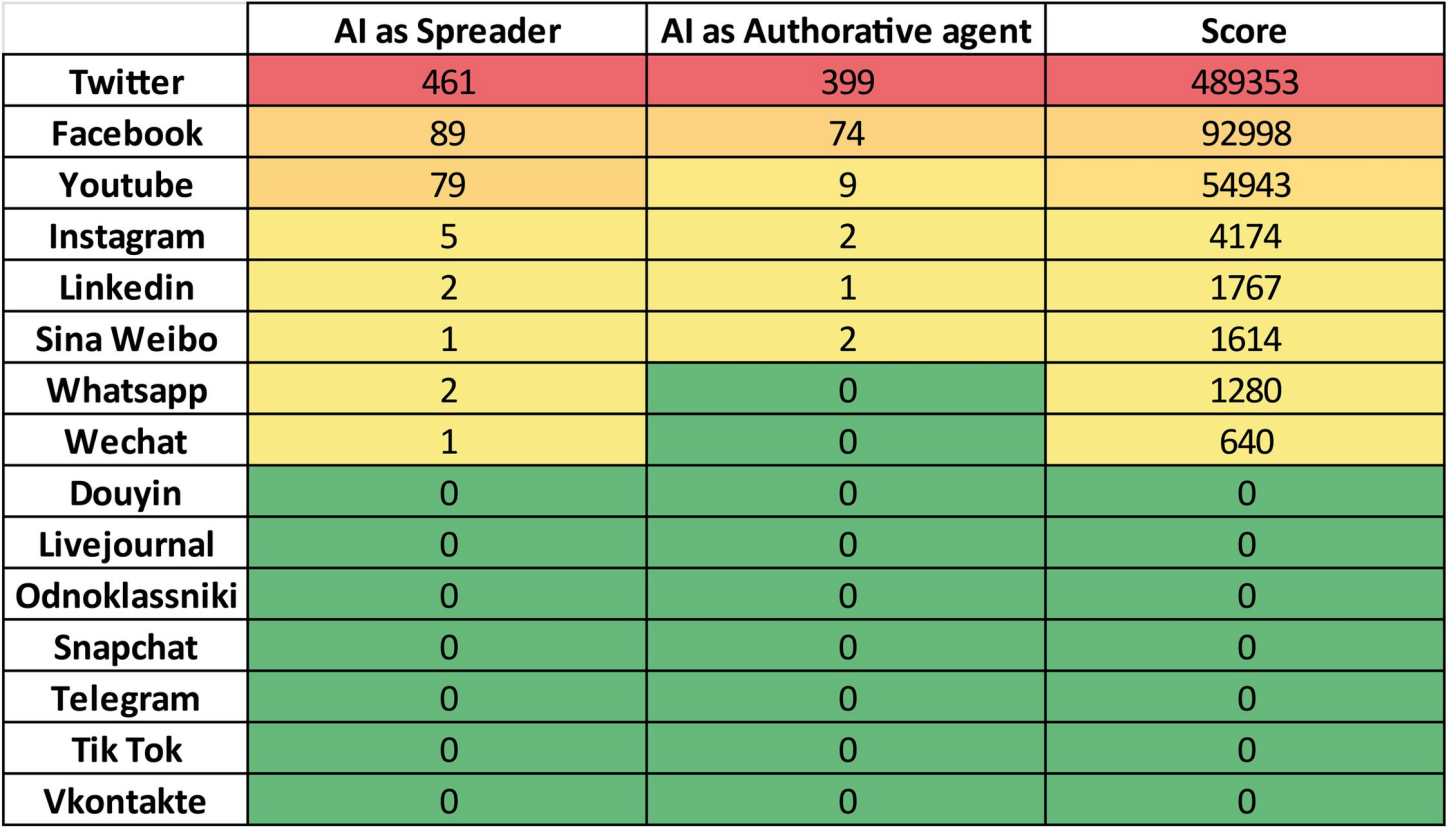
AI bots vs social media platform. Correlation matrix between AI behavior addressed in the screened papers and the most widely used social networks worldwide.

In this literature review, several studies have been screened to explore the role of artificial intelligence (AI) in the dissemination of fake news. Surprisingly, the findings reveal that AI acts as both a spreader of fake news and an authoritative agent. On one hand, the power of AI can be harnessed to uncover and identify fake news, potentially aiding users in distinguishing between genuine and fabricated information [340,341].On the other hand, AI can also serve as a harmful agent, amplifying and spreading false or incorrect information, thereby posing a significant challenge in accurately assessing the authenticity of news sources [33,342]. These contradictory findings highlight the complexity and potential pitfalls associated with relying on the sole AI for the analysis of news authenticity. Further research and innovative approaches are required to mitigate the negative impact of AI in spreading fake news and to develop effective mechanisms for its verification. In Fig 15 is shown AI’s behavior versus the dissemination of ID through social networks.

In most cases, AI shows to act more as a spreader than as an authoritative agent. This applies mainly to the most commonly used social networks (Twitter, Facebook, and YouTube). Values are less significant for the less used platforms in the dissemination of news in general (i.e., Instagram, Linkedin, Sina Weibo, etc.), and is not present at all in the papers analyzed coming from those social platforms reported with a green background.

**Fig 16:**
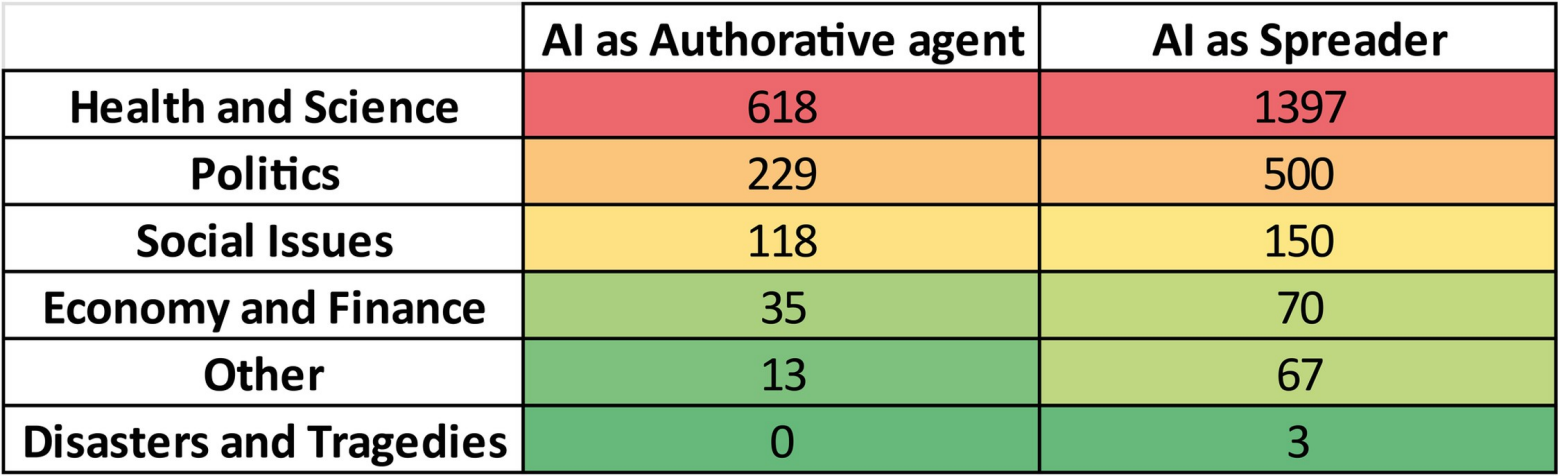
AI bots vs topics. Correlation matrix between AI behavior addressed in the screened papers and the major topics of dissemination of the ID.

It’s also interesting to consider the specific areas or topics in which AI operates as a disseminator of fake news or as an authoritative agent (see Fig 16).

This analysis too shows that AI behaves more like a fake news spreader instead of an authoritative agent used to protect users from IDs. This finding is due to several factors. First, AI algorithms rely heavily on data and patterns, often without fully understanding the nuances of context and credibility [343]. If it aligns with popular narratives or generates high engagement, it can lead to the unintentional amplification of misleading or false information. Additionally, the algorithms used by AI systems may prioritize maximizing user attention and interaction rather than prioritizing accuracy and authenticity [344]. This can result in the promotion of sensationalized or controversial content, including fake news, as it tends to generate more clicks, likes, and shares. Furthermore, the ever-evolving nature of fake news makes it challenging for AI systems to consistently and effectively identify and combat them. The manipulation tactics employed by purveyors of fake news continue to evolve, often surpassing the capabilities of AI systems designed to detect them. Consequently, the limitations of AI in accurately discerning between genuine and false information contribute to its tendency to inadvertently spread fake news instead of acting as a reliable authoritative agent.

### Empowering critical thinking in tackling fake news

The role of Critical Thinking in the papers analyzed in this literature review is essential and multifaceted. Critical Thinking serves as a crucial tool in combating the detrimental effects of fake news by encouraging individuals to question, analyze, and evaluate the information they encounter [345,346]. It can alleviate the fear and panic that false alarms and sensationalized headlines can trigger, promoting a more rational and measured approach to news consumption [347,348]. Critical Thinking also aids in mitigating the impact of consensus bias, wherein individuals tend to believe information that aligns with their preexisting beliefs or the prevailing narrative [349]. By fostering a mindset of skepticism and inquiry, Critical Thinking helps to counteract narrative division and confusion by promoting a more nuanced understanding of complex issues [350,351]. Additionally, Critical Thinking acts as a shield against the allure of clickbait, which often leads to the spread of misinformation. By empowering individuals to assess the credibility and reliability of sources, Critical Thinking mitigates the distress and panic caused by ID [349,352]. Overall, the scientific literature recognizes that, although it often requires triggers to be activated [353], Critical Thinking as an essential component in navigating the landscape of fake news and its detrimental consequences, offering a potential solution to combat its spread and protect individuals from its harmful effects.

It is also important to note that Critical Thinking plays a crucial role in addressing fake news, as relying on the sole AI as a trained critical thinker on behalf of the user is not effective enough [354]: there are inherent limitations to AI systems that prevent them from effectively emulating the nuanced cognitive processes involved in Critical Thinking [355,356]. First, AI lacks the ability to grasp the intricacies of human emotions, values, and biases, which are essential components in critically evaluating information [357,358]. Critical Thinking requires an understanding of the broader context, cultural nuances, and the ability to discern subjective intent, factors that AI struggles to accurately interpret. AI systems primarily rely on algorithms and data patterns, which can be manipulated or biased themselves, leading to potential inaccuracies and reinforcing existing biases. Additionally, AI algorithms are not equipped to adapt and evolve at the same pace as the ever-changing tactics employed by those spreading fake news [343,344]. The dynamic nature of fake news necessitates human judgment and reasoning, which AI currently falls short of replicating. Therefore, while AI can assist in certain aspects, it cannot replace the inherent cognitive abilities of human critical thinking when it comes to detecting and combating fake news effectively.

Understanding the fragilities of the human mind is crucial to fully harnessing the potential of AI. By recognizing the limitations and biases that humans possess, we can better leverage AI as a complementary tool in the fight against fake news. By combining the strengths of AI, such as its ability to analyze vast amounts of data and detect patterns, with human critical thinking skills, we can create a more robust system for identifying and countering fake news. This approach acknowledges that AI can aid in information processing, fact-checking, and identifying inconsistencies, but it requires human judgment to interpret the findings and consider the broader context. By bridging the gap between human cognition and AI capabilities, we can maximize the potential of both effectively combating fake news and protecting users from its detrimental effects.

## Discussions and conclusions

### Navigating the information landscape: Partisan bias and fact-checkers

The issue of fake news on social media is a pressing concern with significant implications. While social media platforms have implemented measures to combat the spread of misinformation, it is evident that partisan bias can still influence fact-checking efforts [359]. Researchers have made efforts to study this phenomenon by creating data repositories that provide insights into the spread of fake news on social media [341,360–362]. However a different dimension to the issue is highlighted by pointing out that anti-critical thinking practices can be detrimental to the development of critical thinking skills [363,364]. Such practices can limit free speech, suppress dissenting opinions, and promote misinformation, which can hinder the understanding of complex topics [365,366]. Therefore, it is essential to address the issue of anti-critical thinking to ensure that individuals develop the necessary skills to navigate the complex information landscape of social media.

*Partisan bias* refers to the tendency of people to interpret or report information in a way that is consistent with their political beliefs or affiliations [367,368]. In the context of fact-checking efforts on social media platforms, partisan bias can influence the way in which information is evaluated and classified as true or false [369]. For example, if a fact-checker has a political bias toward a particular party or ideology, they may be more likely to label information that corresponds with their beliefs as true and information that contradicts their beliefs as false. This can lead to a situation where misinformation is labeled as true or facts are labeled as false, which can further exacerbate the problem of fake news on social media [370]. Therefore, it is essential to mitigate the impact of partisan bias on fact-checking efforts to ensure that the information provided is accurate and unbiased. One example of how partisan bias has affected fact-checking efforts is the controversy surrounding Facebook’s program on third-party fact-checking [371,372]. In 2019, it was revealed that some of the fact-checkers hired by Facebook had political biases that influenced their decisions. For example, one of the fact-checkers, who was affiliated with a conservative think tank, was found to have labeled true posts from left-leaning sources as false, while false posts from right-leaning sources as true. This led to accusations of bias and raised concerns about the effectiveness of Facebook’s fact-checking program. Similarly, in 2020, Twitter received criticism for labeling a tweet from a conservative commentator as "manipulated media," while tweets with similar content from left-leaning sources were left unchecked [373]. These examples illustrate how partisan bias can influence fact-checking efforts and highlight the need for more rigorous and transparent fact-checking processes to combat the spread of misinformation on social media.

It can be challenging for users to identify fact-checkers with political biases, as these biases may not always be apparent [374]. However, there are some steps that users can take to evaluate the credibility of fact-checkers and the sources they use [375]. First, users can check the credentials of the fact-checkers to determine if they have expertise in the relevant area. Secondly, users can examine the sources cited by the fact-checkers to determine if they are reputable and unbiased. Additionally, users can compare the fact-checkers’ conclusions with those of other fact-checkers to see if there is a consensus. Finally, users can look for any evidence of political biases in the fact-checkers’ work, such as consistently labeling posts from a particular political ideology as false or true. However, it’s important to note that identifying political biases in fact-checkers can be a difficult task, and users should be cautious when evaluating the credibility of fact-checkers and the information they provide. There are several ways to determine if a source is reputable and unbiased:

- Check the author or organization behind the source: Look for information about the author or organization to see if they have a reputation for producing accurate and unbiased information. You can do this by searching for the author or organization on search engines or checking their website.
- Look for other sources to corroborate the information: Check other sources to see if they are reporting the same information. If multiple sources are reporting the same information, it is more likely to be accurate.
- Check the date of the source: Make sure that the source you are using is current and up-to-date, as information can become outdated quickly.
- Check for bias: Look for any signs of bias in the source, such as a clear political or ideological agenda. If the source appears biased, it may not be the most reliable source of information.
- Pay attention to the tone of the source: Look for any emotional language or inflammatory statements that could indicate bias or an agenda.

By considering these factors, is possible to get a better sense of whether a source is reputable and unbiased. However, it is important to remember that no source is completely unbiased, and it is always a good idea to check multiple sources to get a more comprehensive understanding of a topic [376]. It’s important to approach the information with caution: if it is impossible to find any corroborating sources or additional information, it may be best to withhold judgment or refrain from using the information until more reliable information becomes available [377].

### Visualizing information: How a knowledge graph can streamline your data management

In today’s information-saturated world, the volume of available knowledge presents a significant challenge. Traditional taxonomic structures, such as Linnaean trees or encyclopedias, are no longer sufficient to navigate this complex landscape. Additionally, the direct verification of reliable sources has become increasingly difficult. To address this issue, we propose an organizational framework derived from a comprehensive review. This framework aims to systematize and simplify knowledge organization, providing a solution to the overwhelming influx of information. By adopting this systematic approach, we can effectively manage and navigate the vast sea of information that surrounds us. In the realm of image recognition and cognitive processes, the utilization of cognitive artifacts, such as knowledge graphs, can greatly enhance cognitive capacities. Cognitive artifacts are tools or objects that assist in performing cognitive tasks more efficiently and accurately [378,379]. Knowledge graphs, structured representations of knowledge, offer a powerful cognitive artifact for enhancing image recognition capabilities. By organizing and capturing information about visual concepts, knowledge graphs facilitate a deeper understanding of visual information [380,381]. These tools prove to be valuable in representing and containing a huge amount of information and allow them to be navigated to grasp interesting findings and connections. They enable the comprehensive representation of both the topics and the social networks addressed in the analyzed papers, fostering a holistic understanding of these domains. Knowledge graphs not only provide an efficient means of representing complex relationships between concepts but also facilitate the discovery of new patterns and relationships [382]. Additionally, they can recommend personalized pathways based on topic interests or use of social networks, improving the dataset exploitation experience. Knowledge graphs allow a *simplex management* method [383] of literature review playing a crucial role in streamlining data management and overcoming the issue of *information silos* [384,385]. Information silos refer to the isolated storage and limited accessibility of information within specific domains or disciplines. This can hinder interdisciplinary collaboration and impede the comprehensive understanding of complex topics [384]. Simplex management allows to overcome the challenges posed by information silos, enabling the integration of diverse sources creating a unified holistic and interconnected knowledge base view of the research field [383]. The simplex management approach involves the systematic organization and synthesis of literature to extract key insights and findings. By consolidating information from various sources, simplex management enables researchers to navigate the vast amount of literature and identify relevant studies more effectively [383]. Combining the power of knowledge graphs and simplex management results in a streamlined and comprehensive approach to data management. The knowledge graph serves as a visual representation of information, facilitating the exploration and understanding of complex relationships. Simultaneously, simplex management ensures the systematic organization and synthesis of literature, preventing the fragmentation of knowledge and enabling a more cohesive and informed research process. These characteristics can greatly enhance cognitive capacities and streamlining data management, and a deeper understanding of information belonging to complex domains. Consequentially, researchers can navigate the vast amount of information more efficiently and uncover new insights.

### Unraveling the dynamics of fake news through literature

The prevalence of fake news and its impact on individuals’ beliefs requires a comprehensive understanding of the underlying communication processes. This study delves into the intricate stages involved in the dissemination of false information, emphasizing the crucial need to understand the factors that contribute to individuals’ susceptibility to misleading content. Particularly in scenarios where false beliefs can lead to adverse outcomes, unraveling the mechanisms behind belief formation becomes imperative. Notably, the landscape of fake news propagation has evolved, with a growing shift towards closed social media applications. Within these closed networks, fake news effortlessly traverses from sender to receiver, concealing itself from the scrutiny of those outside the conversation. This hidden transmission poses significant challenges in combating misinformation and underscores the urgency of comprehending its dynamics.

[386] states falsehoods diffuse considerably faster and more broadly than truths on Twitter. The study analyzed over 126,000 Twitter stories tweeted by about 3 million people more than 4.5 million times and found that false political news had more pronounced effects than false news about less-partisan topics such as terrorism, natural disasters, science, urban legends, finance, or health issues, such as COVID-19 pandemic information. This study provides information on the growing trend of accessing news and information through social technologies, more precisely an increasing proportion of adults prefer to get their news online, including through social media platforms. The paper also discusses how AI can be used to detect and combat fake news on social media and the ethical concerns surrounding the use of AI in detecting fake news. AI algorithms can be also used for "dark creativity" to generate emotionally-loaded fakes for profit and notoriety. Such systems with explicitly deceptive intentions put AI technology at a disservice to society. Moreover, there are concerns about the potential biases in AI algorithms that could lead to false positives or negatives in detecting fake news. Humans are not always good at distinguishing between real and fake news, especially when the content aligns with their pre-existing beliefs or biases. This is known as *confirmation bias*. Additionally, humans may not have the time or resources to fact-check every piece of information they encounter online. AI can be used to complement human abilities in detecting fake news and improving overall accuracy amplifying and complementing human critical thinking by mimicking the procedures and know-how of experts or by requiring entirely new systematic approaches. Additionally, AI can be used to assist humans in detecting fake news by providing additional information and context that may not be immediately apparent to humans. However, it is important to note that AI should not replace critical thinking skills but rather enhance them.

According to [387] some examples of misinformation spreading on social media include rumors and unverified information shared during breaking news situations. For instance, after a terror attack on the Champs Élysées in Paris in April 2017, individuals on social media unwittingly published rumors, such as the news that a second policeman had been killed. People sharing this type of content are rarely doing so to cause harm but are caught up in the moment and fail to adequately inspect and verify the information they are sharing. The authors mention that various third-party actors have created websites that use a set of criteria to fact-check trending online content or certify the credibility (trustworthiness) of popular online news websites. Social media platforms have begun fact-checking what is posted and shared on their sites by users. However, the jury is still out on how vigorously and successfully they do this. As for reporting misinformation on social media platforms, most platforms have reporting features that allow users to flag content as false or misleading.

[388], in the paper titled "Creating Chaos Online" argues that the impact of disinformation on a society as a whole can be significant. Disinformation can render publics vulnerable to propaganda and influence attitudes and behaviors in target populations. Anonymity and automation are two factors that can contribute to the proliferation of disinformation on online platforms. Anonymity allows users to assume masked or faceless identities, which can make it easier for them to generate posts on news portals or social networking sites without being held accountable for their actions. Similarly, automation can foster the amplification and proliferation of disinformation by allowing certain ideas or information to spread rapidly from the margins to the mainstream. This can occur through the use of AI, bots, and other automated tools that are designed to amplify certain messages or content. These factors can make it easier for disinformation campaigns to gain traction online and reach a wider audience than they might otherwise be able to. Anonymity and automation are both typical features of the sociotechnical structure of online platforms. The term "sociotechnical" refers to the interplay between social and technical factors in shaping the design, use, and impact of technology. In the case of online platforms, the sociotechnical structure includes both the technical features of the platform (such as its algorithms, user interface, and data architecture) as well as the social practices and norms that emerge around its use (such as how users interact with each other, what types of content are shared, and how information is evaluated). Anonymity and automation are two examples of technical features that can have significant social consequences. By enabling users to remain anonymous or by amplifying certain types of content over others, these features can shape how information is produced, circulated, and consumed on online platforms. As a result, understanding the sociotechnical structure of online platforms is crucial for understanding how disinformation spreads online and what can be done to address it. According to the aforementioned article "Creating Chaos Online," disinformation tactics used online can include the deployment of propaganda that involves affective, deflective, and misleading information. The work also notes the recurrence of justification frames, which are similar to disinformation propaganda tactics of past and present dictatorships.

[389] discuss about the concept of polarization. This concept refers to the phenomenon where people with similar beliefs and values become more extreme in their views after taking position. This can lead to a widening gap between different groups in society, as each group becomes more entrenched in their own beliefs and less willing to consider alternative perspectives. Polarization can be influenced by various factors, including media consumption, social networks, and political discourse. Empirical studies have shown that blogs and personalized news portals can contribute to political polarization in society. In the USA, for example, supporters of the Republican Party have moved further to the right in recent years, while Democrats have drifted further to the left. The paper also covers topics that contribute to shaping opinions by polarization and societal divisions, including the transformation brought by the Internet, the influence of search engines like Google, the role of blogs and social media platforms. All these factors lead to the analysis of the power of framing and narratives, the creation of filter bubbles and echo chambers through social media algorithms, and the detrimental effects of conspiracy theories. Overall, Zoglauer’s article underscores the erosion of trust in traditional sources of authority and calls for critical examination of beliefs and open dialogue to foster a more nuanced understanding of truth.

[390], the "One-Dimensional Discourse" is analyzed. This is a concept that refers to limited communication characterized by a lack of critical thinking and analysis, reinforcing dominant ideologies and power structures. It is associated with authoritarianism, consumerism, and technological progress, leading to the colonization of human experience. Social media, considered a "new communicative paradigm," enables various forms of electronic communication and content production. However, within the context of communicative capitalism, social media can foster one-dimensional discourse by capturing resistance and promoting capitalist ideals. This plays a significant role in shaping public discourse and influencing political opinions. Moreover, the impact of social media on communication is analyzed, highlighting its transformative nature and potential for reinforcing dominant ideologies and power structures, ultimately affecting public discourse and political opinions.

In "Optimising Emotions, Incubating Falsehoods," by [391] practical strategies are provided to protect against disinformation and misinformation, such as fact-checking and critical thinking. Disinformation is intentionally spread to deceive, while misinformation may be spread without deceptive intent. The book highlights real-life examples of the impact of false information on global events, including the rise of populist movements and its influence on political elections and public health. It also discusses deepfakes and shallowfakes, manipulated videos that misrepresent reality. The dynamics of false information online involve the economics and politics of emotion, optimizing emotional content for financial and political gain. The authors emphasize the scale and virality of false information, involving bots and various types of spreaders that use emotionalized presentation to amplify their reach.

In [392] the authors discuss the relationship between fake news, conspiracy theories, and digital media. They argue that conspiracy theories are a dangerous form of fake news facilitated by the affordances of the digital media ecology. Conspiracy theorists not only believe in these theories but also generate content to spread them. The authors also highlight the emergence of fake news in the past few years, causing public anxiety and debates on truth, media responsibility, and audience literacy. They connect fake news to postmodern culture, where spectacle triumphs over substance, truth becomes relative, and reality is constructed through media representations. The authors draw parallels between fake news and propaganda, suggesting a similar impact on Donald Trump’s election. They emphasize the challenge posed by deepfake videos, which masquerade as authentic and manipulate viewers in an era of hyperreality and disinformation.

In "Building Back Truth in an Age of Misinformation," [393] the author emphasize the importance of being *critical* consumers of media to identify reliable sources. This involves evaluating source credibility, checking for bias, and verifying information with other sources. Social media platforms have accelerated the spread of false information, rewarding pages that share misinformation with more engagement. These platforms often evade responsibility as publishers. Educators play a crucial role in teaching students to combat misinformation by evaluating sources and incorporating critical media skills into the curriculum. Designers and developers can create healthier online communities by implementing features like limiting visibility of likes and shares, providing context for posts, promoting diverse perspectives, and reducing anonymity to discourage harmful behavior.

## Conclusions: Illuminating insights and future directions

In conclusion, this scientific literature review analysis delved into the phenomenon of information disorder on social media platforms, with a particular focus on the dissemination of fake news related to politics, health, and science. Our findings shed light on the distinct ways in which misinformation, disinformation, and malinformation spread across various platforms, with Twitter being a common platform for political propaganda and Facebook for health-related misinformation. We also emphasized the dual role of artificial intelligence in both perpetuating and combatting false narratives. To combat information disorder, we proposed several strategies, including enhancing digital literacy skills and fostering critical thinking among social media users. However, it is important to acknowledge the limitations of our review, as it is based solely on scientific literature and may not encompass all aspects of the phenomenon. Moreover, the rapid pace of social media makes it challenging to keep up with the latest trends in fake news. Moving forward, future research should explore innovative approaches to tackling information disorder on social media platforms. Leveraging emerging technologies such as blockchain and machine learning algorithms could offer promising avenues to verify the authenticity of information. Additionally, concerted efforts should be made to promote digital literacy skills and encourage critical thinking to empower users in navigating the online information landscape. In conclusion, our review contributes fresh insights into the intricate issue of information disorder on social media platforms and presents potential solutions to address this pressing concern. By fostering collaboration and continuing research in this field, while harnessing the power of knowledge graph simplexity data management techniques, we can foster a more informed and responsible digital society. While we have identified several strategies for combating fake news, there are limitations to our review. For instance, our analysis is limited to the scientific literature and may not capture all aspects of the phenomenon. Additionally, the fast-paced nature of social media makes it difficult to keep up with the latest trends in fake news. Our study assumes that the extent of informational disorders on social media and AI bot behaviors is accurately reflected in the volume of scientific articles on these topics. This assumption becomes more credible as the number and recency of relevant articles increase. However, this approach has limitations due to the scientific literature’s potential lag in capturing the rapid evolution of digital behaviors. Factors such as publication bias and the academic community’s response time to emerging trends could affect the comprehensiveness of our analysis. Thus, while our methodology provides a substantial basis for understanding these phenomena, it necessitates cautious interpretation of findings, acknowledging the possibility of underrepresentation or delayed recognition of new developments in social media and AI bot activities. An additional limitation of the study concerns the exclusive use of the Scopus database for identifying articles relevant to our review. Although Scopus is renowned for its broad coverage and the high quality of indexed publications, it does not capture the entire spectrum of scientific publications. This approach has the potential to omit relevant studies published outside Scopus. However, given Scopus’s high coverage percentage in our specific research domain and the inclusion of major influential works, we believe that this limitation does not significantly compromise the robustness and representativeness of the results obtained. Future research could extend the analysis to additional databases to compare results and assess the impact of this methodological choice on the overall understanding of the field. Moving forward, future research should explore new ways to combat information disorder on social media platforms. One potential avenue is to leverage emerging technologies such as blockchain or machine learning algorithms to verify the authenticity of information. Furthermore, efforts should be made to promote digital literacy skills among users and encourage critical thinking when consuming information online.

## Supporting information

S1 File (XLSX)
